# An Attention‐Aware Multi‐Task Learning Framework Identifies Candidate Targets for Drug Repurposing in Sarcopenia

**DOI:** 10.1002/jcsm.13661

**Published:** 2025-03-05

**Authors:** Md Selim Reza, Chuan Qiu, Xu Lin, Kuan‐Jui Su, Anqi Liu, Xiao Zhang, Yun Gong, Zhe Luo, Qing Tian, Martin Nwadiugwu, Shaung Liang, Hui Shen, Hong‐Wen Deng

**Affiliations:** ^1^ Deming Department of Medicine, School of Medicine, Tulane Center for Biomedical Informatics and Genomics Tulane University New Orleans Louisiana USA; ^2^ Shunde Hospital of Southern Medical University Foshan China; ^3^ Central South University Changsha China

**Keywords:** aging biomarkers, canagliflozin, drug repurposing, multi‐omics, sarcopenia

## Abstract

**Background:**

Sarcopenia presents a pressing public health concern due to its association with age‐related muscle mass decline, strength loss and reduced physical performance, particularly in the growing older population. Given the absence of approved pharmacological therapies for sarcopenia, the need to discover effective pharmacological interventions has become critical.

**Methods:**

To address this challenge and discover new therapies, we developed a novel **M**ulti‐**T**ask **A**ttention‐aware method for **M**ulti−**O**mics data (MTA−MO) to extract complex biological insights from various biomedical data sources, including transcriptome, methylome and genome data to identify drug targets and discover new therapies. Additionally, MTA‐MO integrates human protein–protein interaction (PPI) networks and drug‐target networks to improve target identification. The novel method is applied to a multi‐omics dataset that included 1055 participants aged 20–50 (mean (± SD) age 36.88 (± 8.64)), comprising 37.82% African‐American and 62.18% Caucasian/White individuals. Physical activity levels were self‐reported and categorized into three groups: ≥ 3 times/week, < 3 times/week and no regular exercise. Mean (± SD) measures for grip strength, appendicular lean mass (ALM), exercise frequency and smoking status (no/yes, *n* (%)) were 38.72 (± 8.93) kg, 28.65 (± 4.63) kg, 4.31 (± 1.79) and 30.81%/69.19%, respectively. Significant differences (*p* < 0.05) were found between groups in age, ALM, smoking, and consumption of milk, alcohol, beer and wine.

**Results:**

Using the MTA‐MO method, we identified 639 gene targets, and by analysing PPIs and querying public databases, we narrowed this list down to seven potential hub genes associated with sarcopenia (*ESR1*, *ATM*, *CDC42*, *EP300*, *PIK3CA*, *EGF* and *PTK2B)*. These findings were further validated through diverse levels of pathobiological evidence associated with sarcopenia. Gene Ontology and KEGG pathways analysis highlighted five key functions and signalling pathways relevant to skeletal muscle. The interaction network analysis identified three transcriptional factors (*GATA2*, *JUN* and *FOXC1*) as the key transcriptional regulators of the seven potential genes. In silico analysis of 1940 drug candidates identified canagliflozin as a promising candidate for repurposing in sarcopenia, demonstrating the strongest binding affinity to the PTK2B protein (inhibition constant 6.97 μM). This binding is stabilized by hydrophobic bonds, Van der Waals forces, pi‐alkyl interactions and pi‐anion interactions around PTK2B's active residues, suggesting its potential as a therapeutic option.

**Conclusions:**

Our novel approach effectively integrates multi‐omics data to identify potential treatments for sarcopenia. The findings suggest that canagliflozin could be a promising therapeutic candidate for sarcopenia.

## 1 Introduction

Sarcopenia is an age‐related disease that manifests as a gradual decline in skeletal muscle mass and strength, coupled with diminishing physical function [1]. It is linked to the loss of skeletal muscle fibres and muscle atrophy. Aging individuals grappling with sarcopenia face elevated risks of adverse outcomes, which include frailty, diminished quality of life, falls, fractures, disability and even mortality [2]. The prevalence of sarcopenia increases with age, affecting roughly 10% of people aged 60–70 years and rising to 30% for individuals over 80 [2]. As the population ages, sarcopenia is becoming a major public health concern. In the US alone, the estimated cost of sarcopenia in 2000 was a hefty $18.5 billion, representing 1.5% of all healthcare spending [3], and the economic burden has only grown since then [4]. Despite increased research into potential therapies for sarcopenia, exercise remains the primary treatment, and notably, no drug has yet been approved by the FDA for this condition [5]. Treatment success rates for sarcopenia is constrained by the limited understanding of the diverse genetic characteristics associated with the disease [6], which is affecting many people worldwide [7]. Therefore, there is the need to create sustainable treatment options for people with sarcopenia.

Multi‐omics data can offer deeper insights into the biological mechanisms underlying complex traits and disorders, which have a significant impact on global health [2, 8, 9]. These frequently arise from the complex interactions between multiple layers of omics data [10, 11]. A single‐omics approach can only detect changes in a limited segment of the biological pathway, potentially missing many biomarkers of complex diseases [11]. Integrating various types of biomedical data is a major challenge in modern research. According to the findings by Chaudhary et al., early integration where all data sets are merged before analysis [12] have limitations [13]. Firstly, it concentrates the weight on datasets with an abundance of features. Secondly, it overlooks distribution differences among datasets. Lastly, it increases the model's input dimension. To address these issues, late integration models have emerged as a solution, tackling each dataset independently before amalgamating them [14], to maintain the distinct distribution of data. However, during the integration stage, weak signals in any dataset could be lost [13], and it is also possible that the biological relationships between features across different datasets will be lost [14]. Recently, an advanced supervised multi‐modal and ‐omics machine learning (ML) integration framework (MOMLIN) was developed to improve the prediction of anticancer drug responses [15]. However, this method may struggle with scalability and computational complexity, making it less efficient for large datasets. That is why it is crucial to develop a novel, interpretable method for identifying robust aging biomarkers through multi‐omics data to deepen our comprehension of the complex factors affecting drug responses.

To overcome limitations in current methods, we present a **M**ulti−**T**ask **A**ttention‐aware approach for **M**ulti−**O**mics data (MTA‐MO) to identify robust aging biomarkers for drug repurposing. This innovative and adaptable approach is designed to extract complex biological insights from various biomedical data, with the goal of achieving better performance. We employed a geometric method to convert genes and modules into numerical representations (vectors). The vector sum of genes within each module is treated as the module vector, marking a novel use of gene and module vectorization in deep neural networks (DNNs). MTA‐MO incorporates an attention mechanism inspired by Moon et al., which serves as a mediator [16]. This mechanism acts like a bridge, identifying interrelated modules across multiple datasets. The result is an intermediate integration strategy that effectively addresses the drawbacks of both early and late integration methods. This approach preserves the unique distribution of each dataset, considers important biological interactions and avoids undue increases in the input dimension of the model.

We applied MTA‐MO to analyse our generated multi‐omics dataset from the Louisiana osteoporosis study (LOS) data to identify key factors associated with sarcopenia. To validate our findings, we employed a comprehensive range of analyses, including protein–protein interaction (PPI) network analysis to select top‐ranked hub genes (HubGs); enrichment analysis to explore key transcription factors (TFs) of HubGs; GeneMANIA prediction analysis for predicting the biological function of HubGs; GeneHancer analysis for retrieving comprehensive information about HubGs, including their function, expression and related diseases; and molecular docking analysis to identify repurposable drugs for sarcopenia. Our results showed that MTA‐MO outperforms both ML and deep learning (DL) methods in predicting lean muscle mass. Additionally, our findings highlight canagliflozin as a promising candidate for drug repurposing in sarcopenia treatment.

## 2 Materials and Methods

### 2.1 Dataset

The Louisiana osteoporosis study (LOS) is an ongoing cross‐sectional research project that started recruiting participants in 2011. Its goal is to gather a large sample group of around 20 000 peoples to create a database for studying the genetic and environmental factors related to sarcopenia, osteoporosis, obesity and other complex diseases. Peoples aged 18 and older from Baton Rouge, New Orleans, and nearby areas in Louisiana were invited to participate. The specific criteria for who could join and who had to be excluded can be found in previous publications [17, 18]. We analysed data from 1055 participants who were randomly selected males within the age range of 20 to 50 years and provided complete survey responses as well as valid measurements. Before collecting any data, we obtained consent forms from all participants, and the study was approved by the Tulane University Institutional Review Board.

#### 2.1.1 Measurements

##### 2.1.1.1 Body Composition, Anthropometrics and Grip Strength

The dual‐energy X‐ray absorptiometry (DXA) machine (Hologic QDR‐4500 Discovery DXA scanner, Hologic Inc., Bedford, MA, USA) was used to measure body composition, including whole body fat mass, whole body lean mass, per cent fat mass (% fat mass) and appendicular skeletal lean mass, by trained and certified research staffs. The machine undertook daily calibration, and both software and hardware were regularly updated during data collection. The lean mass phenotypes derived using manufacturer's image analysis protocols as demonstrated elsewhere [19], were used in the following analyses. Participants did not need to fast before undergoing DXA scans. Height and weight were measured while wearing light indoor clothing and no shoes, using a calibrated stadiometer and balance beam scale, respectively. Appendicular lean mass (ALM) was determined by summing the non‐fat mass and non‐bone mass of the four limbs, while the skeletal muscle index (SMI) was calculated as ALM divided by height squared [20]. Grip strength of both hands was measured twice using a hand‐held dynamometer (TEC Inc., Clifton, NJ) with the maximum value recorded in kilograms. Sarcopenia‐related traits were assessed using whole body lean mass, SMI and grip strength [20].

##### 2.1.1.2 Physical Activity

Physical activity levels were assessed by asking participants if they exercised regularly and how often they exercised per week. The frequency of self‐reported exercise was divided into three groups: exercising 3 or more times a week, 1 to 2 times a week, and no regular exercise. These measures have been used in various scientific publications [21, 22]. According to the Physical Activity Guidelines for Americans 2nd edition, engaging in physical activity at least 3 days a week provides significant health benefits [23]. Even smaller amounts of activity, such as 1 to 2 times a week, are beneficial compared to being inactive. Physical activity encompasses any movement that burns energy, while exercise is a type of planned, structured and repetitive activity aimed at improving or maintaining physical fitness [24].

##### 2.1.1.3 Demographics and Other Covariates

Questionnaire survey was used to gather information such as age, gender, race/ethnicity, menopausal status, and other behaviours linked to body composition indicators [S1–S4]. Participants indicated their race/ethnicity from options including African American/Black, Asian, Caucasian/White, Hispanic/Latino, Native American/Pacific Islander and other. Responding ‘yes’ to the question ‘Are you postmenopausal?’ was considered as post‐menopause. Other behaviours were assessed with yes/no questions about smoking, alcohol use, milk consumption (including fortified soy, rice and almond milk), calcium supplements and whether they had about 15 min of daily sun exposure. The main characteristics of the participants are presented in Table 1.

**TABLE 1:** The main characteristics of the participants.

Characteristics	Total (*n* = 1055)	Caucasian/White (*n* = 656)	African‐American (*n* = 399)	*p*
Age (years), mean ± SD	36.88 ± 8.64	35.46 ± 8.73	39.23 ± 7.98	**4.14E‐12**
Height, mean ± SD	175.11 ± 6.96 (cm)	175.29 ± 6.90 (cm)	174.81 ± 7.05 (cm)	0.26911
Weight, mean ± SD	83.08 ± 16.78 (kg)	83.24 ± 16.23 (kg)	82.82 ± 17.65 (kg)	0.68911
Grip strength, mean ± SD	38.72 ± 8.93 (kg)	38.91 ± 8.52 (kg)	38.37 ± 9.63 (kg)	0.47131
LARM_LEAN, mean ± SD	3.92 ± 0.70 (kg)	3.81 ± 0.68 (kg)	4.11 ± 0.69 (kg)	**7.95E‐12**
RARM_LEAN, mean ± SD	4.02 ± 0.72 (kg)	3.90 ± 0.71 (kg)	4.22 ± 0.71 (kg)	**4.81E‐12**
L_LEG_LEAN, mean ± SD	10.33 ± 1.69 (kg)	10.10 ± 1.57 (kg)	10.74 ± 1.81 (kg)	**8.06E‐10**
R_LEG_LEAN, mean ± SD	10.40 ± 1.70 (kg)	10.19 ± 1.56 (kg)	10.79 ± 1.84 (kg)	**6.31E‐09**
WBTOT_LEAN, mean ± SD	63.51 ± 9.40 (kg)	63.12 ± 9.06 (kg)	64.15 ± 9.90 (kg)	0.08292
ALM, mean ± SD	28.65 ± 4.63 (kg)	27.96 ± 4.52 (kg)	29.85 ± 5.04 (kg)	**2.89E‐11**
Smoke (no/yes, *n* (%))	30.81%/69.19%	34.15%/65.85%	25.31%/74.69%	**0.00255**
Milk drinking (no/yes, *n* (%))	18.39%/81.61%	16.31%/83.69%	21.80%/78.20%	**0.02489**
Alcohol drinking (no/yes, *n* (%))	34.03%/65.97%	26.68%/73.32%	46.12%/53.88%	**2.19E‐10**
Exercises weekly, mean ± SD	4.31 ± 1.79	4.29 ± 1.75	4.32 ± 1.86	0.81402
Sun exposure (no/yes, *n* (%))	12.89%/87.11%	12.65%/87.35%	13.28%/86.72%	0.77421
Beer drinking (no/yes, *n* (%))	47.77%/52.23%	41.62%/58.38%	57.89%/42.11%	**2.52E‐06**
Wine drinking (no/yes, *n* (%))	62.65%/37.35%	57.47%/42.53%	71.18%/28.82%	**3.23E‐05**
Economic level^a^	1 (*n* = 453), 2 (*n* = 144), 3 (*n* = 59), 4 (*n* = 20), 5 (*n* = 41), NA (*n* = 338)	1 (*n* = 273), 2 (*n* = 116), 3 (*n* = 54), 4 (*n* = 17), 5 (*n* = 37), NA (*n* = 159)	1 (*n* = 174), 2 (*n* = 30), 3 (*n* = 8), 4 (n = 3), 5 (n = 4), NA (*n* = 180)	

Abbreviations: ALM, appendicular lean mass; LARM_LEAN, lean tissue in the left arm; RARM_LEAN, lean tissue in the right arm; L_LEG_LEAN, lean tissue in the left leg; R_LEG_LEAN, lean tissue in the right leg; and WBTOT_LEAN, total lean tissue in the whole body.

^a^ The economic levels (personal annual income) were categorized into under $20 000, $20 000–$39 999, $40 000–$59 999, $60 000−$79 999 and $80 000 or more, which were coded as 1, 2, 3, 4 and 5, respectively.

#### 2.1.2 Omics Data Generation (Bioinformatics Analysis) and Preprocessing

The whole genome sequencing (WGS) for single‐nucleotide variants (SNV), whole genome bisulfite sequencing (WGBS) for DNA methylation (meth) profile, and RNA sequencing for gene expression (mRNA) profile were performed and provided the detailed procedure in the Supporting Information: Sections S1.1–S1.4. Cell deconvolution analysis and data preprocessing procedure are provided in the Supporting Information: Sections S1.5 and S1.6.

### 2.2 Overview of the Framework

**FIGURE 1:**
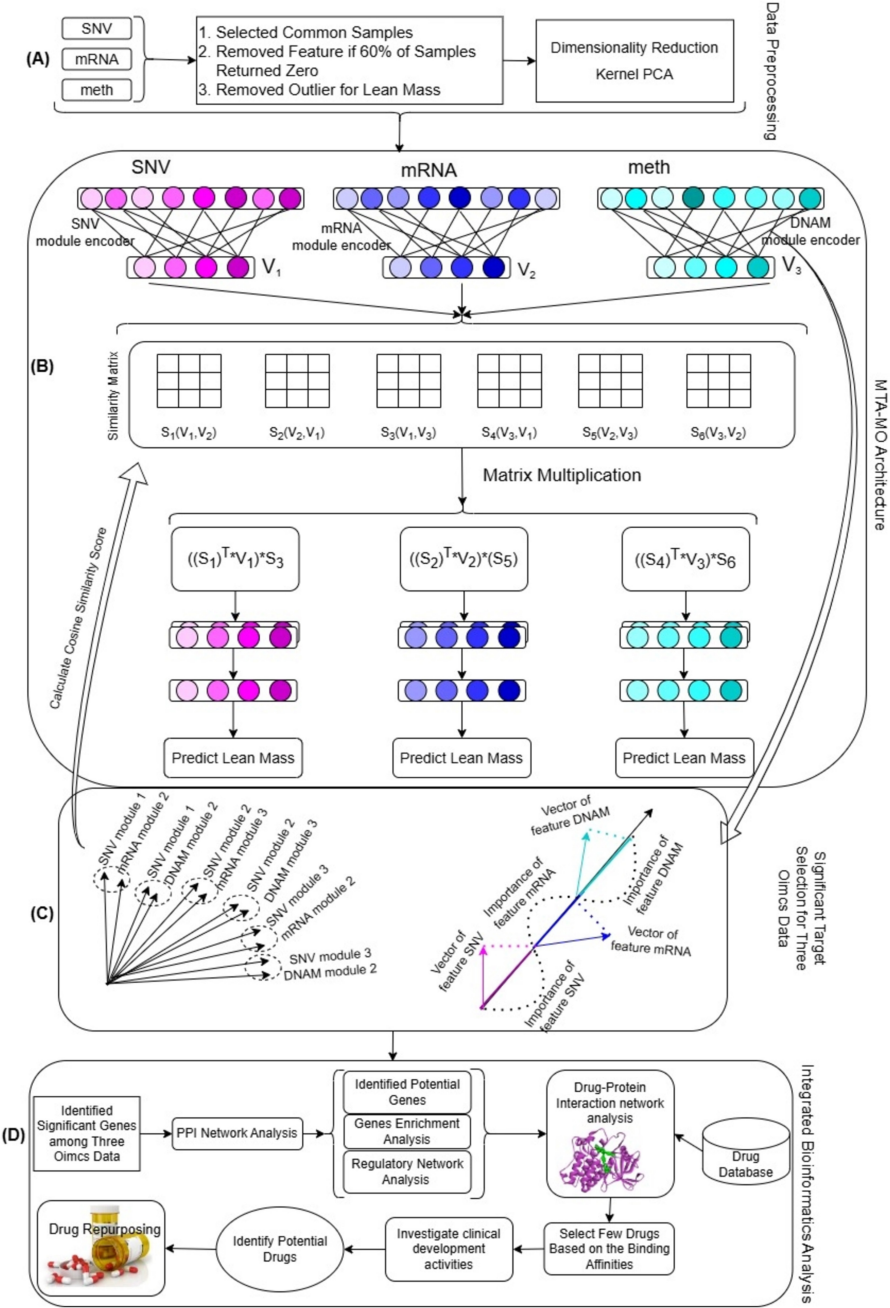
An overview of the MTA‐MO model for integrating biomedical multi‐omics data to identify potential therapeutic targets and treatments for sarcopenia. (A) Data preprocessing for three omics datasets:SNV, mRNA and meth. (B) The MTA‐MO model consists of three main steps: (1) creating a module for each dataset through the module encoder, (2) emphasizing crucial modules across different omics data using module attention, and (3) employing multi‐task learning for each dataset within a fully connected layer. The relationship between genes and modules is represented by the weights of the fully connected layer, and the weight vectors of individual genes show the weights between each gene and each module. The gene vector is formed by multiplying the input value of the gene with its weight vectors. The module vector is created by adding up the vectors of all genes within that module. (C) The degree of gene contribution to the module vector is represented by the inner product of a gene vector and a module vector. The vectors for SNV, mRNA and meth modules are input into an attention layer, which gives greater importance, or higher weights, to the module vectors that are particularly relevant for predicting sarcopenia variables. (D) Screening potential drug agents based on in silico study.

We proposed the MTA‐MO model for multi‐omics module analysis to identify target genes for drug repurposing in sarcopenia. Our model consists of three main steps: (1) creating a module for each dataset through the module encoder, (2) selecting crucial cross‐omics modules across different omics data using module attention, and (3) employing multi‐task learning for each dataset within a fully connected layer (see Figure 1B,C). The way we have structured the MTA‐MO model to integrate multi‐omics data and predict variables related to sarcopenia is outlined below.

#### 2.2.1 Module Encoder

The module encoder is made up of a fully connected layer that links features from omics data to each module, with a vector representing each module. Consider a training sample {$x^{m}$, *z*; *m* = 1, 2, 3}, where $x^{m}$ represent the sample under different omics profiles (SNV, mRNA and meth), and z is the corresponding lean mass variables. The weights of the fully connected layer, denoted as $w_{\text{module}}^{i}$, represent the connection between features and modules of the *m*th omics data. Each weight vector corresponds to a feature and a module, indicating their associations. The output nodes from the fully connected layer are normalized into unit vectors, representing module vectors. Let us denote the module encoder as $f_{\text{module}}^{m}$ of *m*th omics data and the module vector for *m*th omics data $V^{m}$ are defined as follows:

(1) $$\begin{array}{c} V^{m}\left(x^{m}\right)=f_{\text{module}}^{m}\left(x^{m};W_{\text{module}}^{m}\right)\in {\mathbb{R}}^{N^{m}\times D}, \end{array}$$

where $f_{\text{module}}$ is made up of a fully connected layer and unit vector normalization, with $W_{\text{module}}$ representing the weights of $f_{\text{module}}$. D representing the dimension of the module vector, and $N^{m}$ refer to the number of modules of *m*th omics data.

#### 2.2.2 Module Attention Mechanism

We created a module attention mechanism to concentrate on modules that exhibit high similarity across each omics data module. To assess relevance, cosine similarity was employed. Let us use S to represent the module similarity matrix between the module vectors of two omics datasets, and $S_{\mathrm{lk}}$ to denote the element at the intersection of row l and column k (specifically, the (*l*, *k*)th element of S). $S_{\mathrm{lk}}$ contains relationship information indicating potential interdependence between the *l*th module from one omics dataset and the *k*th module from another omics dataset. The similarity matrices were denoted as follows:

(2) $$\begin{array}{c} S_{\mathrm{lk}}^{p}\left(V^{n},V^{m}\right)=\frac{\exp\left(\cos\left(V_{l}^{m},V_{k}^{n}\right)\right)}{\sum_{K=1}^{N^{m}}\exp\left(\cos\left(V_{l}^{m},V_{k}^{n}\right)\right)},\\ \;s.t.\;m,n\in \left\{1,\ldots ,m\right\},m\neq n \end{array}$$

where $\;V^{m}=V^{m}\left(x\right)$ for short, $V_{l}^{n}$ and $V_{k}^{m}$ are denotes *l*th module vector of *n*th omics data and *k*th module vector of *m*th omics data, respectively. Each element of $S_{\mathrm{lk}}$ stores the relation information with possible dependence between the *l*th module from one dataset and the *k*th module from another dataset module. *p* denotes the number of all possible combination for three omics datasets. Modules with high similarity to the other two data sets were focused and used for prediction. In order to emphasize the crucial modules, we multiply the module vectors with similarity matrices from other omics data and then combine them. The resulting module vector is then updated as follows:

(3) $$\begin{array}{c} U\_V^{m}\left(x^{m}\right)=\left[{\left(S^{q}\left(V^{m},{\bar{V}}^{m}\right)\right)}^{T}\times V^{m}\times S^{p}\left(V^{m},{\bar{V}}^{m}\right)\right],\\ \;s.t.\;p,q\in \left\{1,\ldots ,p\right\},p\neq q,\\ \;\text{and}\;{\bar{V}}^{m}\in \left\{V|V\neq V^{m}\right\} \end{array}$$

where $V^{m}=V^{m}\left(x^{m}\right)$ for short and *m* represents the number of omics datasets. To learn more about how the attention mechanism works in this module, please see the Supporting Information: Method S1.1.

#### 2.2.3 Other Importance Methods

The details of the other methods we used in this study were provided in the supplementary file. The training strategy, important feature selection through our model, and experimental settings are thoroughly explained in Supporting Information: Sections S2.3–S2.5, respectively. The comprehensive network analysis of significant genes is provided in Supporting Information: Sections S2.6–S2.10.

## 3 Results

### 3.1 Sarcopenia Data Analysis

We applied MTA‐MO framework to analyse our generated multi‐omics dataset from the LOS data (details in Section 2.1). The dataset is accessible at our lab, SNV dataset which included 1010 samples and 23 536 genes, meth dataset which included 985 samples and 23 688 genes, and mRNA dataset which included 944 samples and 19 286 genes, from African‐American; Caucasian and Hispanic males with sarcopenia variables (see Table S1).

In this study, we compared the performance of our model against several baseline ML and DL models, such as convolutional neural networks (CNNs), deep neural networks (DNNs) and XGBoost in terms of mean square error (MSE), root mean square error (RMSE) and mean absolute error (MAE) performance based on various combinations on omics datasets. Table 2 showed the MSE, RMSE and MAE values for our model with several ML and DL models when integrating three omics datasets. MTA‐MO outperformed the others in terms of MSE, RMSE and MAE when using only individual dataset types. For instance, the MSE values for our model were 52.017, 53.436 and 54.003 for the mRNA, meth and SNV datasets, respectively. Similarly, our model demonstrated RMSE values of 7.223, 7.309 and 7.348, and MAE values of 5.515, 6.016 and 6.260 for mRNA, meth and SNV omics datasets, respectively.

**TABLE 2:** Comparison of the prediction performance of the scoring function for three omics datasets.

	MSE	RMSE	MAE
**mRNA**			
XGBoost	56.512	7.517	5.829
CNN	109.827	10.479	6.751
DNN	284.647	16.871	14.174
MTA‐MO	52.017	7.223	5.515
**meth**			
XGBoost	54.691	7.395	5.658
CNN	61.540	7.844	5.853
DNN	285.030	16.882	14.380
MTA‐MO	53.436	7.309	6.016
**SNV**			
XGBoost	55.772	7.468	5.691
CNN	66.712	8.167	5.919
DNN	61.375	7.834	5.652
MTA‐MO	54.003	7.348	6.260

Among the baseline methods, the XGBoost demonstrated the second‐best performance in terms of MSE, scoring 56.512, 54.691 and 55.772 for mRNA, meth, SNV omics datasets, respectively. For the same datasets, the RMSE and MAE of XGBoost were (7.517 and 5.829), (7.395 and 5.658) and (7.468 and 5.691) for mRNA, meth and SNV omics datasets, respectively. On the other hand, the DNN and CNN methods showed worse performance compared to others. Following closely, the CNN method achieved the third‐best performance in terms of MSE, scoring 109.827, 61.540 and 66.712 for mRNA, meth and SNV omics datasets, respectively. Correspondingly, the RMSE and MAE of CNN were (10.479 and 6.751), (7.844 and 5.853) and (8.167 and 5.919) for the same datasets, respectively. On the other hand, we also explored different combinations, like integrating two omics datasets, to compare ALM prediction performance of our method (see Table S5). Our analysis indicated that MTA‐MO outperforms when using three omics datasets compared with two omics datasets. The grid search outcomes for MTA‐MO on the validation sets are detailed in Tables S2, S3 and S4.

### 3.2 Selection of Important Features

We looked into the attention of specific modules to understand which genes are influential in predicting sarcopenia variables across three omics datasets. For each type of data (mRNA, meth and SNV), we identified pairs of module vectors that were most attentive to sarcopenia during training. These pairs had the highest cosine similarity scores in our analysis. We then calculated the importance of genes within these modules using Supporting Information: Equation S12 and calculated their *Z*‐scores. Genes with *Z*‐scores above certain thresholds (set at the 98th, 98.9th and 97th percentiles for mRNA, meth and SNV, respectively) were considered significant. Our analysis identified 269 significant genes for mRNA, 207 for meth and 200 for SNV (see Table S6).

### 3.3 Construct the Protein–Protein Interaction (PPI) Network Analysis of Significant Genes

**FIGURE 2:**
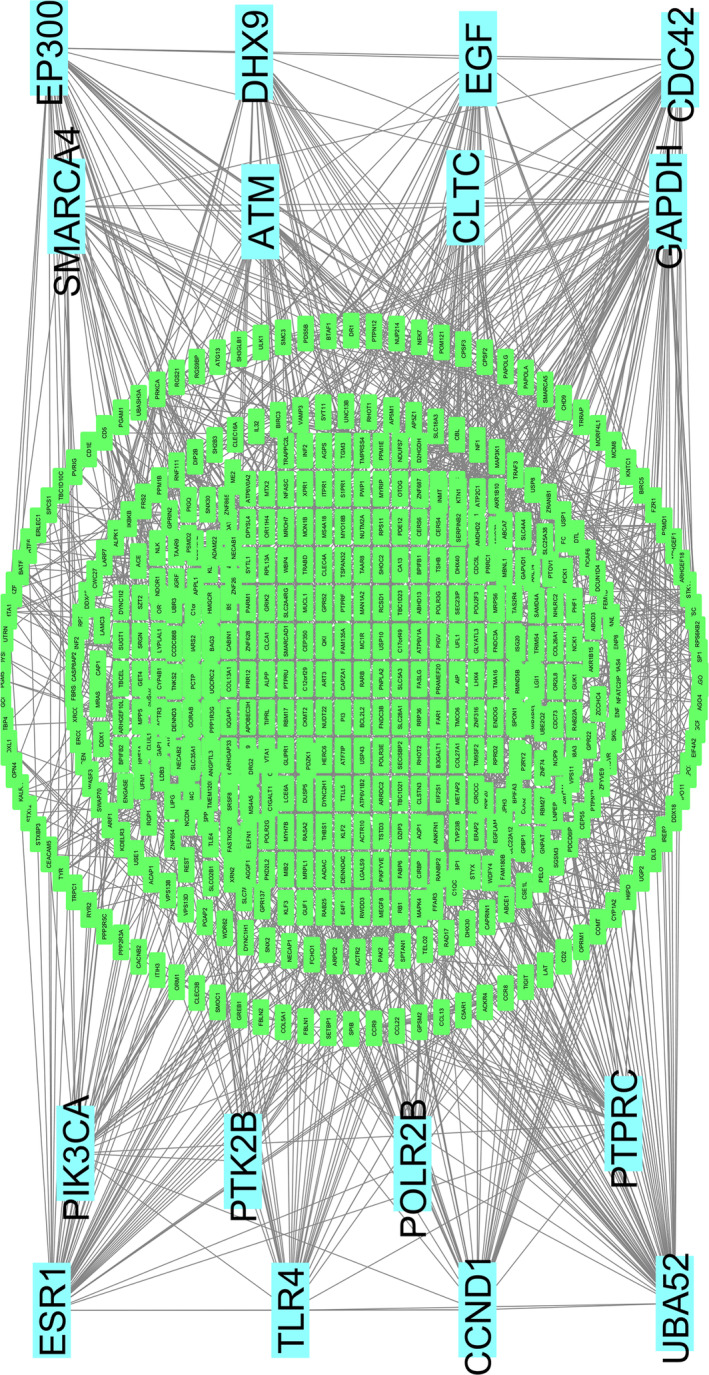
Screening of the significant genes among SNV, mRNA and meth omics datasets. Protein–protein interaction network for the significant genes of sarcopenia, and edges specify the interconnection in the middle of two genes. The analysed network holds 518 nodes and 1747 edges. The highlighted 16 nodes (*ESR1*, *ATM*, *UBA52*, *SMARCA4*, *CDC42*, *EP300*, *PIK3CA*, *CLTC*, *TLR4*, *EGF*, *GAPDH*, *PTPRC*, *POLR2B*, *CCND1*, *DHX9* and *PTK2B*) represented the hub genes (HubGs) for sarcopenia.

After applying our proposed method, we identified 639 unique genes across three omics datasets (see Table S6). Using the STRING database, we constructed a Protein–Protein Interaction (PPI) network with 518 nodes and 1747 edges (Figure 2). The degree method helped us to identify 16 hub genes (HubGs), of which 10 (*ESR1*, *EP300*, *PIK3CA*, *CLTC*, *TLR4*, *GAPDH*, *PTPRC*, *POLR2B*, *DHX9* and *PTK2B*) came from mRNA, 4 (*ATM*, *CDC42*, *CLTC* and *SMARCA4*) from meth, and 4 (*CCND1*, *UBA52*, *SMARCA4* and *EGF*) from SNV data. The network analysis of each gene (Table S7) confirmed more significant interactions than expected. Comparing them to aging data from the GenAge Human Genes database [S5], seven genes (*ESR1*, *ATM*, *CDC42*, *EP300*, *PIK3CA*, *EGF* and *PTK2B*) were identified as aging‐related potential genes (PGs) (Table S8). Further analysis using the GeneCards and GeneMANIA databases showed strong connections among these genes in various biological contexts (Figure S1), linking them to complex diseases (Table S9), including skeletal muscle disorders (For more information, see the Supporting Information: Section of S3.1). Then, we evaluated our model's performance in terms of RMSE for the selected aging biomarkers against XGBoost, which previously showed the best results among ML models. The results indicated that MTA‐MO performed better than XGBoost. Specifically, our model's MSE values for the mRNA, meth, and SNV datasets were 12.017, 12.436, and 12.003, respectively, while XGBoost's MSE values for the same datasets were 13.512, 12.891, and 12.772 (see Table S14). Therefore, our model showed the ability to identify undiscovered genes from multi‐omics datasets.

### 3.4 The Regulatory Network Analysis of Potential Genes for Sarcopenia

The network analysis of PGs with transcriptional factors (TFs) detected top‐ranked three significant TFs (*GATA2*, *JUN* and *FOXC1*) as the key transcriptional regulatory factors for the PGs (see Figure S2). We found *GATA2* as key TFs for four PGs (*EP300*, *PTK2B*, *EGF* and *ESR1*), *FOXC1* for six PGs (*ATM*, *CDC42*, *EP300*, *PIK3CA*, *EGF* and *PTK2B*), and *JUN* for three PGs (*ESR1*, *PTK2B* and *EGF*).

Additionally, we tested our method on another data type involving gene‐TF interaction networks. Using the same parameters, our model showed better performance in predicting ALM compared to the baseline method (see Table S10). More details are available in Section 3.2 of the supplementary file.

### 3.5 Functional Enrichment Analysis of Selected 16 Genes

**FIGURE 3:**
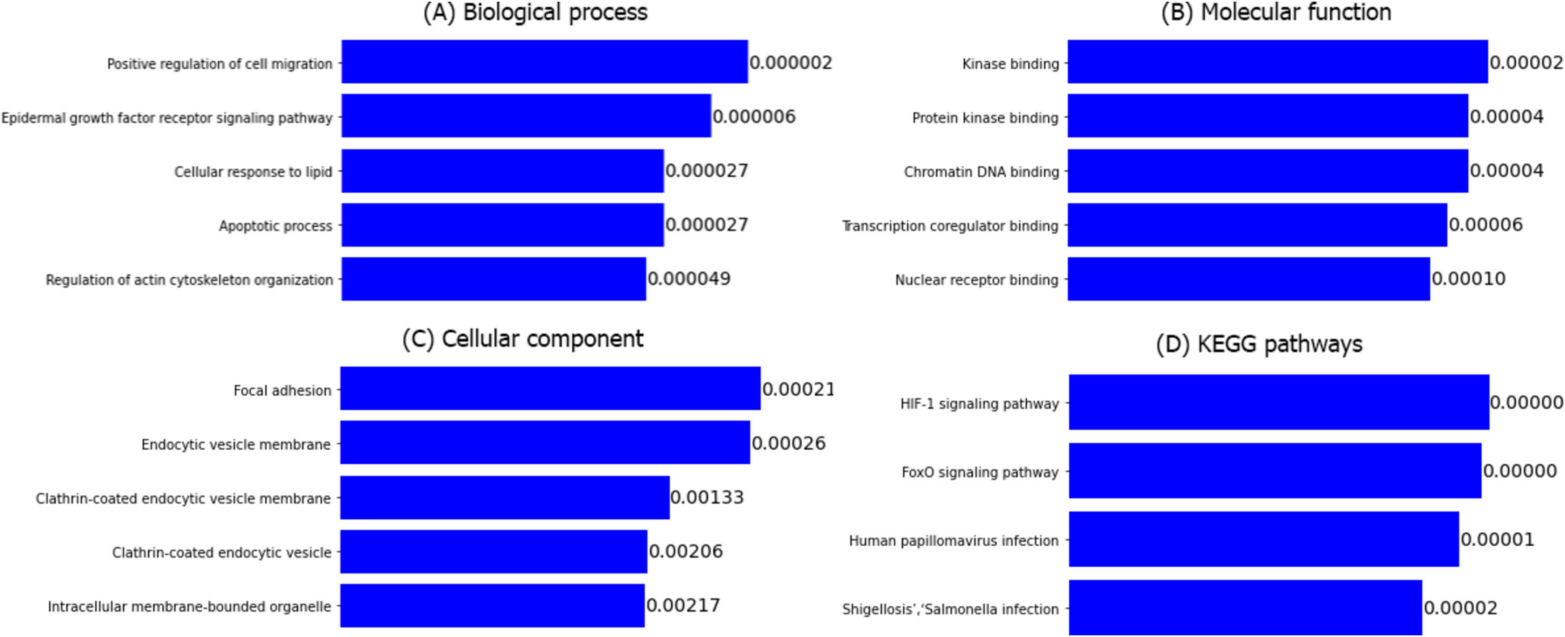
The top 5 significantly (*p*‐value < 0.05) enriched GO functions and KEGG pathways by 16 HubGs with sarcopenia diseases. The significant GO and KEGG terms are displayed in the left side and the significant p‐values are displayed in the right side of each bar. (A) biological process (BP), (B) molecular function (MF), (C) cellular component (CC) and (D) KEGG Pathway.

The GO functional enrichment analysis of DEGs showed that 543 biological process (BP) terms, 52 cellular component (CC) terms and 84 molecular function (MF) terms are enriched by the selected 16 genes, where 7 PGs were involved with 323 BPs, 25 CCs and 48 MFs. On the other hand, 96 KEGG pathways are enriched by the selected 16 genes, where PGs were involved with 89 KEGG pathways (see Figure 3 and Table S11). The top five GO terms of the BPs, including ‘Positive regulation of cell migration’, ‘Epidermal growth factor receptor signalling pathway’, ‘Apoptotic process’, ‘Cellular response to lipid’, and ‘Regulation of actin cytoskeleton organization’ were significantly enriched by the PGs sets *{CDC42*, *EGF*, *PTK2B* and *ATM}*, *{PIK3CA*, *EGF* and *PTK2B}*, *{PIK3CA*, *EP300* and *PTK2B}*, *{PTK2B*, *ATM* and *ESR1}* and *{CDC42*, *PIK3CA* and *PTK2B}*, respectively. We provided the additional GO terms and KEGG pathways in the supplementary file; please see Section 3.3.

### 3.6 Drug Screening Through Docking Study

This study used molecular docking simulations to identify potential drugs for sarcopenia. We collected 1940 drugs from different databases like DrugBank [S6] (https://go.drugbank.com), DGIdb [S7] (https://www.dgidb.org/downloads), and ZINC (https://zinc.docking.org/substances/subsets/fda/), and conducted docking with seven key protein receptors to determine their binding affinities (kcal/mol). From this, we highlighted four top‐performing drugs:Testosterone, MK‐0773, Vorinostat, and Canagliflozin. Then, we investigated the clinical development activities of these four drugs (see Table 3).

**TABLE 3:** Identification of actionable drugs for target genes.

Compound name	Formula	Type	Clinical development activities	Description	Target	Action type
Testosterone	C_19_H_28_O_2_	Small Molecule	Approved, Investigational	Testosterone is a steroid sex hormone indicated to treat primary hypogonadism and hypogonadotropic hypogonadism [51]	AR	Agonist
MK‐0773	C_27_H_34_FN_5_O_2_	Small Molecule	Investigational	For the treatment in sarcopenia (loss of muscle mass) [38].	—	—
Vorinostat	C_14_H_20_N_2_O_3_	Small Molecule	Approved, Investigational	Vorinostat is a histone deacetylase (HDAC) inhibitor used for the treatment of cutaneous manifestations in patients with progressive, persistent, or recurrent cutaneous T‐ cell lymphoma (CTCL) following prior systemic therapies [52].	HDAC1	Inhibitor
Canagliflozin	C_24_H_25_FO_5_S	Small Molecule	Approved	Canagliflozin, also known as Invokana, is a sodium‐glucose cotransporter 2 (SGLT2) inhibitor used in the management of type 2 diabetes mellitus along with lifestyle changes including diet and exercise [50].	SLC5A2	Inhibitor

**FIGURE 4:**
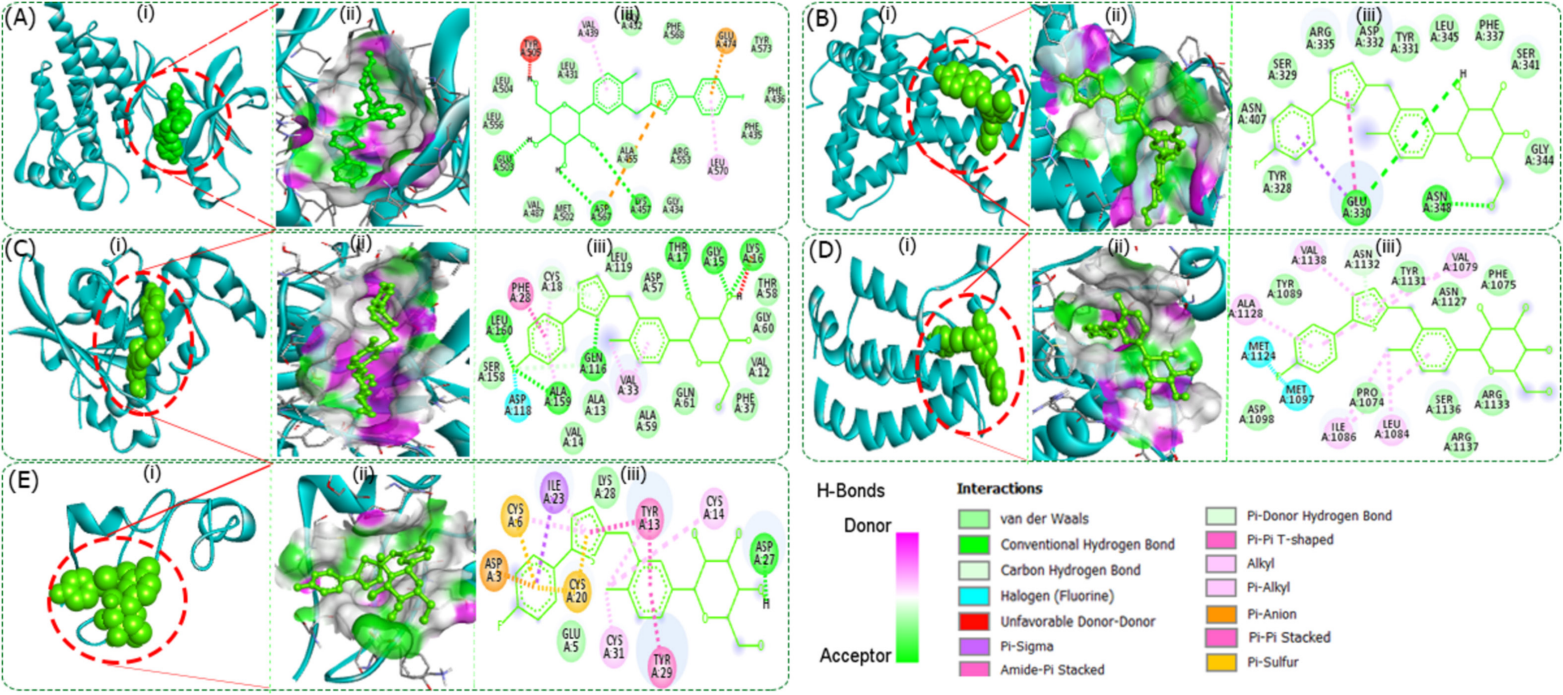
Significant molecular bonding of canagliflozin with five proposed proteins related to sarcopenia. (i) The coupling of canagliflozin with active centre residues. (ii) The enlarged image shows the hydrogen bonds donor and acceptor amino acid residues in the junction cavity. (iii) The 2D plot shows the interaction of the binding pocket residues with the canagliflozin inhibitor. (A) *PTK2B*, (B) *ESR1*, (C) *CDC42*, (D) *EP300* and (E) *EGF.*

Canagliflozin displayed the inhibition constant of 6.97, 6.38, 6.60, 6.09, 6.24, 5.94 and 5.72 (μM) when interacting with *PTK2B*, *ESR1*, *CDC42*, *EP300*, *EGF*, *ATM* and *PIK3CA* proteins, respectively. Our in‐silico analysis revealed that these seven proteins interact with canagliflozin through various types of bonds. These interactions may help in stabilizing the canagliflozin bound with the active residues of human proteins (see Table 4 and Figure 4). Based on the similar binding patterns and docking complex analysis, we can say that canagliflozin is the true, potent inhibitor of these proteins, thus it possibly helps in controlling sarcopenia. Therefore, our in silico findings indicate that canagliflozin could be a promising option for addressing sarcopenia. Please see the Supporting Information: Sections S3.4, S3.5 and S3.6 for comprehensive details.

**TABLE 4:** AutoDock Vina results showing binding energies and inhibition constant of canagliflozin with different proteins related to sarcopenia.

S. no	Protein name (PDB ID)	Theoretical weight (KDa)	Name of chains	Binding energy (ΔG) (kcal/mol)	Predicted inhibition constant pKi (μM)	No. of hydrogen bonds	Hydrogen bonds forming residues
1.	*PTK2B* (3cc6)	35.94	A	−9.5	6.97	3	LYS457, GLU503, ASP567
2.	*ESR1* (1a52)	34.10	A	−8.7	6.38	2	GLU330, ASN348
3.	*CDC42* (1a4r)	24.72	A	−9	6.60	6	GLY15, LYS16, THR17, GLN116, ALA159, LEU160
4.	*EP300* (3i3j)	15.70	A	−8.3	6.09	—	—
5.	*EGF* (2kv4)	7.16	A	−8.5	6.24	1	ASP27
6.	*ATM* (5np0)	407.16	A	−8.1	5.94	3	ASN2267, LEU2270, PRO2271
7.	*PIK3CA* (2enq)	20.44	A	−7.8	5.72	4	LEU99, VAL125, PRO126, GLY128

## 4 Discussion

Sarcopenia is a growing condition that weakens muscles as we age and is linked to an increased risk of illness and death [25]. Despite recent advancements in understanding this condition, there are currently no proven treatments, preventive measures, or reliable indicators (biomarkers) for sarcopenia in clinical practice [26]. Additionally, the underlying causes (pathophysiology) of sarcopenia are complex and not fully understood [27]. To address this challenge, we propose a novel approach for identifying potential treatments for sarcopenia. Our method, called MTA‐MO, integrates various biological data sources (multi‐omics data), protein–protein interactions, and drug–target interactions. We also leverage large public databases to validate potential drug targets and agents. Our research involved a comprehensive analysis of multi‐omics data to identify potential genes associated with sarcopenia. These findings were further validated using various publicly available databases related to human aging. This analysis revealed the involvement of various proteins with different functions, suggesting their potential role in causing sarcopenia. Indeed, our investigation identified canagliflozin as a promising candidate drug for treating sarcopenia. Our findings not only offers new hope for treating sarcopenia but also highlights the power of advanced multi‐omics approaches in understanding complex biological problems.

In this study, we used MTA‐MO methods to analyse our multi‐omics datasets related to sarcopenia. This analysis identified 639 genes that may be relevant to the condition. We narrowed this list down to 16 genes with high potential and explored their functions, cellular pathways, regulatory factors, and potential drug targets. From these, we confirmed seven genes (*PTK2B*, *ESR1*, *CDC42*, *EP300*, *EGF*, *ATM* and *PIK3CA*) possibly related to human aging using GenAge Human database [S5] and considered them as PGs. Then we validated the biological functions of the PGs by utilizing (GeneCards) [S8] and GeneMANIA [S9] which are publicly available databases.

Existing research supports the diverse cellular functions of PTK2B, including bone resorption [S10], neuronal cell process formation [S11], and smooth muscle contraction [28]. Oestrogen is linked to skeletal muscle metabolism, as evidenced by a decrease in muscle ERα mRNA (*ESR1*) levels in women with metabolic syndrome. This decrease is inversely related to adipose tissue mass and fasting insulin levels [29]. The protein *CDC42* plays a crucial role in the regulation of insulin secretion and the aging process [30]. Insights from studies on skeletal muscle cells underscore the pivotal role of p300 in transcriptional regulation during myotube differentiation and maintenance of muscle integrity in vitro [31]. *EGF* inhibitors may be useful in treating the loss of muscle slow‐twitch fibres [32]. The *ATM* protein plays a pivotal role as one of the key regulators and coordinators of the cellular response to DNA damage [33]. We also identified three TFs for PGs and recent research underscores *these TFs are* dysregulation as a potential indicator for skeletal muscle hypertrophy [S12–S14].

The identified 16 significant genes were enriched in many muscle‐related GO terms. Evidence revealed that cell migration plays a crucial role in embryonic development, as well as the repair and regeneration of skeletal muscle, which is the body's most abundant tissue [34]. The epidermal growth factor receptor signalling pathway is crucial for controlling the growth, proliferation, survival, and differentiation of mammalian cells [35]. Apoptotic signalling in muscle tissue is commonly linked to muscle wasting and impaired function [36]. Lipid droplets undergo significant changes as skeletal muscle satellite cells progress through myogenesis [37]. Actin is a fundamental element in the motion of the cytoskeleton, and actin‐binding proteins are known to be important regulators of the health and disorders of skeletal muscle [38]. Hence, these GO terms might play crucial roles in skeletal muscle function.

To investigate potential treatments for sarcopenia, we conducted docking analysis on 1940 candidate drugs and identified the top four promising drugs: testosterone, MK‐0773, vorinostat and canagliflozin. Further investigation revealed that the first three drugs were previously identified by others' findings for the treatment of sarcopenia. For instance, testosterone has been found to have a permissive effect on muscle mass and fibre cross‐sectional area in parabiotic mice [S15]. MK‐0773 is currently under investigation in a clinical trial (NCT00529659) to evaluate its safety and effectiveness in women with sarcopenia (muscle mass loss) [S6]. From our group, Liang et al. verified through in vitro validation that vorinostat showed promise as a potential treatment for sarcopenia [S16].

On the other hand, canagliflozin is used to manage type 2 diabetes mellitus in conjunction with lifestyle changes such as diet and exercise. FDA approved it in 2013 for diabetes management and in 2018 for reducing cardiovascular risk in type 2 diabetes patients [S6]. Recent reports indicated that canagliflozin can help repair ischaemic skeletal muscle in mice by reducing *LARS2* expression in muscle stem cells. Canagliflozin also reduces fat mass and lean mass [39], as Naznin et al. observed that treatment with canagliflozin for 8 weeks led to weight loss in mice, with decreased visceral and subcutaneous fat mass [40]. Canagliflozin, an SGLT2 inhibitor, primarily reduces blood glucose levels by promoting glucose excretion in the urine. This mechanism can indirectly affect muscle metabolism. Stable glucose levels can help maintain energy supply to muscles, potentially preserving muscle mass and function [S17]. Therefore, based on our in silico findings and existing data, it is suggested that canagliflozin may be a promising option for addressing sarcopenia requires further validation.

Despite some novel findings, our study has potential limitations. First, we only compared our model with different ML and DL methods. This decision was based on the fact that current multi‐omics integration methods like MOGONET [S18] and P‐NET [S19] are mainly designed for classification tasks, while MOFA [S20] and MEFISTO [S21] are focused on unsupervised integration. In contrast, our approach was specifically developed to handle regression tasks. Second, we only used seven genes to identify potential repurposable drugs for addressing sarcopenia. Since several recent studies have identified new genes for sarcopenia [S16, S22], we may identify new sarcopenia‐associated genes via other multi‐omics data integration such as genomics, proteomics, metabolomics, metagenomics, phenomics and transcriptomics for sarcopenia in the future. Third, though our protocol identified some well‐known drugs associated with sarcopenia such as testosterone and vorinostat, we acknowledge that experimental validation was not performed for the proposed canagliflozin drugs. Additionally, our omics data were generated based on White and African‐American populations, which limits its generalizability to other ethnic groups. Finally, all novel aging biomarkers need to be validated experimentally (including both in vitro and in vivo) and clinical benefits of drugs must be tested in sarcopenia clinical trials in the future.

## 5 Conclusions

In summary, this research introduced an attention‐aware multi‐task learning approach to discover new therapies for sarcopenia by combining various types of biological data, like multi‐omics data and human protein interactions, and integrates analyses from publicly available tools like GeneMANIA, GeneHancer, and pathway analysis. The research goes beyond simply identifying genes linked to sarcopenia; it also investigates how potential drugs might interact with these genes and evaluates their effectiveness based on the mechanism of action. Our model performed better than several strong ML and DL models during comparison. Upon module analysis, it was shown that the module attention mechanism prioritized modules that were rich in biological pathways associated with sarcopenia. This research provided strong evidence of a practical application of multi‐omics integration techniques in gene detection to discover drugs that could possibly be repurposed for sarcopenia; and we successfully identified canagliflozin as a promising therapy. By combining various biological data sources, this research helps bridge the gap between genetic discoveries and effective treatments, which is a major hurdle in developing therapies for sarcopenia.

## Ethics Statement

This research study involving human subjects complied with the ethical principles outlined in the World Medical Association Declaration of Helsinki ‐ Ethical Principles for Medical Research Involving Human Subjects. Institutional approval from Tulane Institutional Review Board (IRB numbers: 968169 and 184 088) was obtained prior to the commencement of the study. The research adhered to the guidelines and regulations set forth by the overseeing body.

## Consent

All participants involved in this manuscript have provided written informed consent to join the LOS study. Any identifying information has been removed to protect their privacy.

## Conflicts of Interest

The authors declare no conflicts of interest.

## Supporting information


**Figure S1.** The network of predicted related genes of 7 significant genes enriched in GenAge Human Genes database.
**Figure S2.** TFs‐genes interaction network with selected 7 genes. The highlighted green‐colour nodes represent the selected 7 genes, cyan‐colour nodes represent the selected TFs (*GATA2, JUN, FOXC1*), and other pink‐colour nodes represent not selected TFs. The network consists of 50 nodes and 69 edges.
**Figure S3.** Superimposition of re‐docked estradiol‐ESR1 (Green) onto co‐crystallized complex (Blue) in the active site using PyMOL (RMSD = 0.0001 Å).
**Table S1.** Summary of omics data.
**Table S2.** The results of the grid search for CV1 and CV2 on biomedical multi‐omics data lean mass prediction task.
**Table S3.** The results of the grid search for CV3 and CV4 on biomedical multi‐omics data lean mass prediction task.
**Table S4.** The results of the grid search for CV5 on biomedical multi‐omics data lean mass prediction task.
**Table S5.** Comparison of the prediction performance of MTA‐MO for two omics data integration.
**Table S7.** Network analysis of each 16 significant genes using String database.
**Table S8.** The potential evidence and Cytogenetic information of 7 genes related to the human aging process.
**Table S9.** The GeneHancer identifier, GeneHancer score, gene association score, total score, and major‐related diseases of 7 selected genes.
**Table S10.** Prediction performance of our method with Identified TFs.
**Table S11.** The top 20 significantly (*p*‐value < 0.05) enriched GO functions and KEGG pathways by significant 16 genes involving 7 selected with sarcopenia.
**Table S14.** Comparison of the prediction performance of MTA‐MO with XGBoost for the potential genes.
**Table S15.** The drug‐protein interaction score based on molecular docking analysis.

**Table S6.** 639 significant genes selected by proposed method.

**Table S12.** The Food and Drug Administration (FDA) approved drugs list.

**Table S13.** The drug‐protein interaction score based on molecular docking analysis.

## Data Availability

The raw data that support the findings of this study are available upon request from the corresponding author, HWD. A part of the relevant data is available within the paper and its supporting information files.
