# Supplementary material for: An Attention‐Aware Multi‐Task Learning Framework Identifies Candidate Targets for Drug Repurposing in Sarcopenia
Source: J Cachexia Sarcopenia Muscle. 2025 Mar 5;16(2):e13661. doi: 10.1002/jcsm.13661 (PMC11883102; doi:10.1002/jcsm.13661)
Supplement: Supplementary file 1 — Figure S1. The network of predicted related genes of 7 significant genes enriched in GenAge Human Genes database. Figure S2. TFs‐genes interaction network with selected 7 genes. The highlighted green‐colour nodes represent the selected 7 genes, cyan‐colour nodes represent the selected TFs (GATA2, JUN, FOXC1), and other pink‐colour nodes represent not selected TFs. The network consists of 50 nodes and 69 edges. Figure S3. Superimposition of re‐docked estradiol‐ESR1 (Green) onto co‐crystallized complex (Blue) in the active site using PyMOL (RMSD = 0.0001 Å). Table S1. Summary of omics data. Table S2. The results of the grid search for CV1 and CV2 on biomedical multi‐omics data lean mass prediction task. Table S3. The results of the grid search for CV3 and CV4 on biomedical multi‐omics data lean mass prediction task. Table S4. The results of the grid search for CV5 on biomedical multi‐omics data lean mass prediction task. Table S5. Comparison of the prediction performance of MTA‐MO for two omics data integration. Table S7. Network analysis of each 16 significant genes using String database. Table S8. The potential evidence and Cytogenetic information of 7 genes related to the human aging process. Table S9. The GeneHancer identifier, GeneHancer score, gene association score, total score, and major‐related diseases of 7 selected genes. Table S10. Prediction performance of our method with Identified TFs. Table S11. The top 20 significantly (p‐value < 0.05) enriched GO functions and KEGG pathways by significant 16 genes involving 7 selected with sarcopenia. Table S14. Comparison of the prediction performance of MTA‐MO with XGBoost for the potential genes. Table S15. The drug‐protein interaction score based on molecular docking analysis. [file JCSM-16-e13661-s001.docx]

**Supplementary Material**

**An attention-aware multi-task learning framework** **identifies candidate targets for drug repurposing in sarcopenia**

Md Selim Reza^1^, Chuan Qiu^1^, Xu Lin^2^, Kuan-Jui Su^1^, Anqi Liu^1^, Xiao Zhang^1^, Yun Gong^1^, Zhe Luo^1^, Qing Tian^1^, Martin Nwadiugwu^1^, Shaung Liang^3^, Hui Shen^1^, and Hong-Wen Deng^1^*

^1^Tulane Center for Biomedical Informatics and Genomics, Deming Department of Medicine, School of Medicine, Tulane University, New Orleans, Louisiana, USA

^2^ Shunde Hospital of Southern Medical University, Foshan City, Guangdong Province, China

^3^ Central South University, China

Corresponding Author

*hdeng2@tulane.edu

**Supplementary Material and Methods**

**1. Omics data generation (bioinformatics analysis)**

**1.1 Whole genome sequencing (**WGS**) for single-nucleotide variants and copy number variants**

We performed WGS on 7950 blood DNA samples which were extracted from the whole blood using the Gentra Puregene Blood Kit (Qiagen, USA). Concentration and quality of the extracted DNA were assessed using Nanodrop 2000 and the samples were kept at -80 °C until further use. Briefly, 300 ng genomic DNA was used as input. Libraries for WGS were prepared with KAPA DNA LTP library preparation kit (KAPA Biosystem, USA) on Biomek FX Laboratory Automation Workstation (Beckman Coulter, USA). The workflow for library preparation consists of DNA Nanoballs (DNBs) generation through ligation-mediated polymerase chain reaction (LM-PCR), single-strand separation, cyclization, and rolling circle amplification procedures. The WGS was conducted at an average read depth of 15x using DNBSEQ-500TM sequencing technology platform (BGI Americas Corporation, Cambridge, MA, USA), with 350 bp paired-end reads in length. Each sample's cleaned and aligned data was mapped to the human reference genome (GRCh38/hg38) using Burrows-Wheeler Aligner (BWA, v0.7.12) software [1]. For accurate variant calling, we adhered to the recommended Best Practices for variant analysis using the Genome Analysis Toolkit (GATK, v4.0.3) [2][3]. HaplotypeCaller of GATK was employed to identify genomic variations, and the variant quality score recalibration (VQSR) was applied to obtain high-confident variant calls [2][3]. The SNPs and InDels marked PASS in the output VCF file were high-confident variation sets. For SNPs recalibration strategy, we used the following datasets and features to train the model. (a) Training sets: HapMap V3.3, Omni2.5M genotyping array data and highconfidence SNP sites produced by the 1000GenomesProject. (b) Features: coverage (DP), quality/depth (QD), Fisher test on strand bias (FS), odds ratio for strand bias (SOR), read position rank sum test (ReadPosRankSum). The Copy Number Variants (CNVs) were called using the CNVnator v0.3.3 [4] read-depth algorithm. The structural variations (SV) were detected using Breakdancer v1.4.5 [5][6] or CREST [7]. Then the ANNOVAR tool was applied to perform a series of annotations for variants.

The single-nucleotide variation (SNV) frequency for each gene was defined as the number of non-silent SNVs in that gene, divided by the exonic gene length [8]. Non-silent SNVs in the coding regions that give "birth" to amino acid mutations, are often involved in the modulation of protein function. Exonic gene length is the sum of the exon lengths per gene ID (Extract Total Non-Overlapping Exon Length Per Gene).

**1.2 Isolation of Monocytes, Their Genomic DNA, and Total RNA**

In the present study, we focused specifically on peripheral blood monocytes (PBMs), which can act as osteoclast precursors and play important roles in regulating bone metabolism [9][10]. Briefly, peripheral blood mononuclear cells (PBMCs) were firstly separated from ~60 mL freshly collected peripheral blood, by a density gradient centrifugation method using Histopaque-1077 (Sigma-Aldrich, USA). The PBMCs were washed repeatedly with 2 mM EDTA in PBS, before being dissolved in 0.5% BSA and 2 mM EDTA in PBS. PBMs were then isolated from the PBMCs with a Monocyte Isolation Kit II (Miltenyi Biotec Gmbh, Bergisch Glagbach, Germany) according to the manufacturer's protocol. The kit depleted unwanted cells (such as T and B cells) from PBMCs, leaving PBMs free of the surface-bound antibody and beads with minimum disturbance. The isolated PBMs were visually checked for purity and counted under microscope. The genomic DNA used for WGBS and total RNA used for RNA-seq were extracted from the freshly isolated PBMs with the AllPrep DNA/RNA/miRNA Universal Kit (Qiagen, USA) following the manufacturer’s protocol and kept at -80 °C until further use.

**1.3 Whole genome bisulfite sequencing (WGBS) for DNA methylation profile**

DNA methylation profiles were determined by WGBS according to previously published protocols [11]. Briefly, 100 ng genomic DNA isolated from PBMs was fragmented by sonication using a Bioruptor (Diagenode, Belgium) to a mean size of approximately 250 bp, followed by the blunt-ending, dA addition to 3'-end, and adaptor ligation. The ligated libraries were bisulfite converted using the EZ DNA Methylation-Gold kit (Zymo Research Corp, USA).

Data filtering includes removing adaptor sequences, contamination, and low-quality reads from raw reads. Raw reads were also excluded if the number of unknown bases exceeded 10% or if the ratio of bases with a quality score less than 20 exceeded 10%, in order to obtain high-quality and cleaned data. After data filtering, the cleaned reads were mapped to the human reference genome (GRCh37/hg19) using BSMAP [12], which was later converted to GRCh38/hg38 using UCSC LiftOver tool (https://genome.ucsc.edu/cgi-bin/hgLiftOver). The methylation level of a CpG dinucleotide was determined by beta-value, the ratio of the number of methylated reads to the total number of reads covering a particular cytosine site.

Promoter regions were defined as the region within 1000 bp of a transcription start site [8]. The beta values of all CpG sites within the defined promoter window are averaged to calculate the promoter methylation values for each gene. The Beta-value is defined as the ratio of the methylated probe intensity and the overall intensity (sum of methylated and unmethylated probe intensities). For each gene, DNA methylation values were averaged on all samples.

**1.4 RNA-seq for** **gene expression profile**

For the RNA sequencing experiment, RNA integrity was assessed using the Agilent Technologies 2100 Bioanalyzer. Libraries for RNA-seq were prepared following Illumina’s TruSeq-stranded-total-RNA-sample preparation. Briefly, 500 ng RNA was used as input. The workflow consists of rRNA removal, cDNA generation, and end repair to generate blunt ends, A-tailing, adaptor ligation and PCR amplification. Different adaptors were used for multiplexing samples in one sequencing run. The libraries were pooled and diluted to 2 nM in EB buffer and then denatured using the Illumina protocol. The denatured libraries were diluted to 10 pM by pre-chilled hybridization buffer and loaded onto Illumina NovaSeq 6000 sequencing system using a paired-read recipe according to the manufacturer's instructions. Data quality check was done on Illumina SAV. For transcript assembly, Cutadapt (which removes adapter sequences from high-throughput sequencing reads) and in-house Perl scripts were employed to eliminate reads containing adapter contamination, low-quality bases, and undetermined bases. Subsequently, sequence quality was verified using FastQC (http://www.bioinformatics.babraham.ac.uk/projects/fastqc/). Bowtie2 [13] and HISAT2 [14] were utilized to map reads to the human reference genome (GRCh38/hg38). The mapped reads of each sample were assembled using StringTie [15]. Following this, all transcriptomes from all the samples were merged to reconstruct a comprehensive transcriptome using Perl scripts and gffcompare (https://github.com/gpertea/gffcompare/). Upon generating the final transcriptome, StringTie [15] and edgeR [16] were employed to estimate the expression levels of all transcripts. The fragments per kilobase of transcript per million mapped reads (FPKM) for each sample are recorded for each gene. For each gene, RNA expressions were averaged across all samples.

**1.5** **Cell deconvolution analysis**

Cell composition was conducted using the Bayesian cell proportion reconstruction inferred using statistical marginalization (BayesPrism) [17]. Publicly available PBMCs from Healthy Human, Single Cell Gene Expression Profiling Dataset by Cell Ranger v1.1.0, 10x Genomics, (2016, September 29) and PBMCs and Neutrophils, 5’ from Healthy Human, Single Cell Immune Profiling Dataset by Cell Ranger v6.1.0, 10x Genomics, (2021, September 08) were used as the reference in BayesPrism. The reference datasets were clustered using Seurat v5 [18] and visualized using uniform manifold approximation and projection (UMAP, https://arxiv.org/abs/1802.03426). Quality control removed cells with fewer than 200 genes per cell, cells with more than 10,000 counts, and cells with greater than 10% mitochondrial content. Data normalization, scaling, and selection on highly variable genes (HVGs) were performed with SCTransform function in Seurat v5 [18]. Top 3,000 HVGs were selected for downstream analysis, such as dimensional reduction (e.g., principal component analysis (PCA)). The first 30 principal components were obtained and used for dimensionality reduction with UMAP. The two reference datasets were integrated with anchor-based reciprocal PCA (RPCA) integration. Through clustering analysis, we identified 11 clusters and annotated to 6 major cell types: T cells (*CD3*), B cells (*MS4A1*), Natural Killer cells (*GNLY*), dendritic cells (*CD1C, FCER1A, LILRA4*), platelets/megakaryocytes (*PPBP*), CD16+ monocytes (*CD16, MS4A7*) and CD14+ monocytes (*CD14*, *LYZ*). BayesPrism uses the cell type-specific expression profiles from the scRNA-seq reference to estimate the cell type composition in the bulk RNA-seq samples. Then, we adjusted the gene expression profile for CD16+ monocytes and CD14+ monocytes. For WGBS data, we made a common assumption, consistent with other research findings that is the cell proportions derived from RNA-seq data should remain consistent across other omics datasets, given that all biosamples were collected simultaneously from the same individual.

**1.6** **Data preprocessing**

Our process involved meticulously identifying and gathering matched samples from SNV, mRNA, and meth datasets, followed by the systematic elimination of non-informative and noise features inherent in biomedical multi-omics datasets. Any features displaying zero values in more than 60% of the samples were excluded as follows:

$$Used Features in Model= \left\{ \begin{aligned} Keep=Row Sum of Omics\left[ Omics>0 \right] \\ keep\left[ Keep\leq\frac{N}{2} \right];N indicates the number of samples \\ Removed Features, Otherwise \end{aligned} \right.$$

To ensure uniformity, we standardized each omics dataset. Next, employing the robust interquartile range (IQR), we identified and eliminated outliers affecting sarcopenia variables [19]. Each omics dataset is reduced in dimension by applying Kernel Principal Component Analysis (K-PCA), which reduces a large number of characteristics to a smaller number. We utilized K-PCA since it projects a dataset onto a higher dimensional biological feature space, where it is non-linearly separable, using a kernel function.In our model, we included the first five principal components (PCs), which together explain 99% of the variance. Leveraging the versatile KernelPCA package from the sklearn function, we successfully reduced the dimensionality of each omics dataset. This short process not only refines the data but also increases the likelihood of uncovering meaningful insights in our biomedical research.

**2. Supplementary Methods**

**2.1 MTA-MO algorithm on three biomedical omics datasets**

Given a training sample {$x^{1}, x^{2}, x^{3}$, z }, where $x^{1}, x^{2}, {and x}^{3}$ denote the sample under the three omics (gene expression (mRNA), DNA methylation (meth), and Single-nucleotide variation (SNV)) profile, and z is the corresponding appendicular lean mass (ALM) variables. Let $f_{module}$ denotes the module encoder. The module vector of each omics data is defined as follows:

$$V^{1}\left( x^{1} \right)=f_{module}^{1}\left( X^{1};W_{module}^{1} \right)\in\mathbb{R}^{N^{1}\times D}\ldots\ldots\ldots\ldots(1)$$

$$V^{2}\left( x^{2} \right)=f_{module}^{2}\left( X^{2};W_{module}^{2} \right)\in\mathbb{R}^{N^{2}\times D}\ldots\ldots\ldots\ldots(2)$$

$$V^{3}\left( x^{3} \right)=f_{module}^{3}\left( X^{3};W_{module}^{3} \right)\in\mathbb{R}^{N^{3}\times D}\ldots\ldots\ldots\ldots(3)$$

where $f_{module}$ contains of the fully connected layer and unit vector normalization, with $W_{module}$ representing the weights of $f_{module}$. D representing the dimension of the module vector, and $N^{1}, N^{2}, \mathrm{and} N^{3}$refer to the number of SNV modules, the number of mRNA modules and number of meth modules, respectively. Additionally, $V^{1},$ $V^{2}$ and $V^{3}$ represent the module vectors for SNV, mRNA, and meth, respectively.

We created a module attention mechanism to concentrate on modules that exhibit high similarity across each omics data module. To assess relevance, cosine similarity was employed. The similarity matrices were denoted as follows:

$$S_{lk}^{1}\left( x \right)= \frac{\exp\left( \cos\left( V_{l}^{1},V_{k}^{2} \right) \right)}{\sum_{K=1}^{N_{2}} \exp\left( \cos\left( V_{l}^{1},V_{k}^{2} \right) \right)}\ldots\ldots(4), S_{lk}^{2}\left( x \right)= \frac{\exp\left( \cos\left( V_{l}^{2},V_{k}^{1} \right) \right)}{\sum_{K=1}^{N_{1}} \exp\left( \cos\left( V_{l}^{2},V_{k}^{1} \right) \right)}\ldots\ldots(5),$$

$$S_{lk}^{3}\left( x \right)= \frac{\exp\left( \cos\left( V_{l}^{1},V_{k}^{3} \right) \right)}{\sum_{K=1}^{N_{3}} \exp\left( \cos\left( V_{l}^{1},V_{k}^{3} \right) \right)} \ldots\ldots\left( 6 \right), S_{lk}^{4}\left( x \right)= \frac{\exp\left( \cos\left( V_{l}^{3},V_{k}^{1} \right) \right)}{\sum_{K=1}^{N_{1}} \exp\left( \cos\left( V_{l}^{3},V_{k}^{1} \right) \right)} \ldots\ldots(7),$$

$$S_{lk}^{5}\left( x \right)= \frac{\exp\left( \cos\left( V_{l}^{2},V_{k}^{3} \right) \right)}{\sum_{K=1}^{N_{3}} \exp\left( \cos\left( V_{l}^{2},V_{k}^{3} \right) \right)} \ldots\ldots\left( 8 \right), S_{lk}^{6}\left( x \right)= \frac{\exp\left( \cos\left( V_{l}^{3},V_{k}^{2} \right) \right)}{\sum_{K=1}^{N_{2}} \exp\left( \cos\left( V_{l}^{3},V_{k}^{2} \right) \right)}\ldots\ldots\left( 9 \right),$$

where $V=V(x)$for short, $V_{l}$ and $V_{k}$ are denotes l-th and k-th module of the module vector. Each element of $S_{lk}$ stores the relation information with possible dependence between the l-th module from one dataset and the k-th module from another dataset module.

Modules with high similarity to the other two data sets were focused and used for prediction. In order to emphasize the crucial modules, we multiply the module vectors with similarity matrices from other omics data and then combine them. The resulting module vector is then updated as follows;

$$U\_V^{m}\left( x^{m} \right)=\left[ \left( S^{q}\left( V^{m}, \bar{V}^{m} \right) \right)^{T}{\times V}^{m}\times S^{p}\left( V^{m}, \bar{V}^{m} \right) \right], s.t. p,q\in\left\{ 1,\ldots,6 \right\}, p\neq q, and \bar{V}^{m}\in\left\{ 1,\ldots3 \right\},\ldots(10)$$

**2.****2 Training strategy**

Next, we use fully connected layers that transform the multi-dimensional vectors into a flat structure, providing the final probabilities for each ALMs. In the model, the loss (denoted as L) is computed by taking the mean of the squared differences between the empirical measurement and task-specific outputs:

$$L= \frac{1}{M}\sum_{m=1}^{M} \sum_{c=1}^{C} MSE\left( z_{c}, \left( f_{f_{c}}^{m}\left( U\_V^{m}\left( x^{m} \right);W_{f_{c}}^{m} \right) \right) \right)+\alpha\sum\delta^{2}, s.t \delta\epsilon\left\{ W_{module},W_{f_{c}}^{m} \right\}\ldots\ldots\ldots(11)$$

where MSE is the mean squared error between the empirical measurement of lean mass and task-specific outputs. C represents the total number of ALMs, $z_{c}$ denotes the empirical measurement of c, $f_{f_{c}}^{m}$consists of multiple fully connected layers for m-th omics data, $W_{f_{c}}^{m}$ denotes the weights of $f_{f_{c}}$, $\delta$ denotes the weights, and an L2-norm penalty with a regularization parameter $\alpha$ was used for optimization to avoid overfitting the module encoders and the multiple connected layers.

**2.****3 Importance features selection through our model**

The suggested model is a clear and comprehensible one that can predict not just ALM variables but also identify sets of genes associated with sarcopenia variables. The module similarity matrix helps find the most crucial modules for specific ALM variable. This matrix contains cosine similarity values between pairs of module vectors, derived from individual omics datasets. During training, the pair of modules with the highest cosine similarity attracts the most attention for the given ALM variable, making them selected as most applicable modules.

We first identify the most relevant modules and then proceed to choose the crucial features. Let's denote $x_{b}^{m}$ as the value of the b-th feature in the m-th omics data, $W_{module}^{m}(b,d)$ as the weight vector between feature b-th and d-th module, and $V_{d}^{m}\left( x^{m} \right)$ as the d-th module vector of the m-th omics data. Consequently, the feature vector of gene b is obtained by multiplying $x_{b}^{m}$ and $W_{module}^{m}(b,d)$. The significance of the feature within the module is determined by the inner product of the feature vector and the module vector. For instance, the importance of feature b in module d is defined as follows:

$$Importance\left( b,d \right)= \left( x_{b}^{m}\times W_{module}^{m}(b,d) \right)\times V_{d}^{m}\left( x^{m} \right), \ldots\ldots\ldots\ldots\ldots\ldots\ldots\ldots(12)$$

We choose features for each module based on their importance, considering only those that surpass a threshold z value.

**2.****4 Experimental settings**

The models were trained using nested cross-validation to get unbiased performance estimates. Hyperparameters were determined by running inner cross-validation loops within each outer cross-validation loop. Using a 5-fold outer cross-validation setup, we assessed the ALMs prediction performance based on mean square error (MSE), root mean square error (RMSE), and mean absolute error (MAE).

MSE calculates the average of the squared differences between the experimentally determined ALMs and predicted ALMs.

$$MSE= \frac{1}{N}\sum_{i=1}^{N} \left( {Pred}_{LM}-{Real}_{LM} \right)^{2}, \ldots\ldots\ldots\ldots\ldots\ldots\ldots\ldots\ldots\ldots\ldots(13)$$

Where, N represents the number of samples of biomedical omics datasets (SNV, mRNA and meth). ${Real}_{S}$ and ${Pred}_{S}$ indicates the real and predicted ALM, respectively.

RMSE is simply the square root of the MSE. It is useful because it quantifies the relative deviations of the predicted LMs from the experimentally determined ALMs.

$$RMSE= \sqrt{MSE},\ldots\ldots\ldots\ldots\ldots\ldots\ldots\ldots\ldots\ldots\ldots\ldots.(14)$$

MAE is another useful evaluation measurement. Different from RMSE, MAE is the average of the summed absolute differences of the prediction ALMs to the empirical measurement of ALMs.

$$MAE= \frac{1}{N}\sum_{i=1}^{N} \left| {Pred}_{LM}-{Real}_{LM} \right|, \ldots\ldots\ldots\ldots\ldots\ldots\ldots\ldots\ldots..(15)$$

Each model's hyperparameters were determined through 3-fold inner cross-validation on the training set, with the RMSE used as the evaluation metric. Specifically for our model, the parameters for the number of modules were selected from the set {32, 64, 128}, the learning rate from {0.00005, 0.000005, 0.0000005}, the weight decay from {0.0, 0.01, 0.0001}, and the early stopping patience from {50, 100}. The number of fully connected layers was set based on the number of modules. All hidden layers employed ReLU activation, and the final layer used linear functions. In the MTA-MO model, we utilized the adam optimizer [20]. The algorithm details for this experiment and the list of additional model hyperparameters are outlined in supplementary file 1.2 section. The grid search outcomes for MTA-MO on the validation sets are detailed in Supplementary **Table S2, Table S3, and Table S4**.

**2.5 Hyperparameters list**

We proceeded to performance measurements under the 5-fold outer cross-validation (CV). Given data were split into training and test data with a 4:1 ratio. In the training data, the optimal hyperparameters of the model were determined using grid search by inner 3-fold CV in training data. Table S3, Table S3, and Table S5 showed the grid search results for MTA-MO on our in-house datasets.

Hyperparameters are as follows for each model. For XGBoost, the parameters of ‘the max depth’ from the set {3, 5, 7}, ‘the regularization lambda’ from the set {10, 1, 0.1, 0.01}, and ‘the learning rate’ from the set {0.2, 0.1, 0.01} were optimized. For DNN, the parameters of ‘the number of layers’ from the set {16, 32, 64}, ‘the learning rate’ from the set {0.0005, 0.00005, 0.000005}, ‘the weight decay’ from the set {10^−2^, 10^−4^, 10^−6^, 0}, and ‘the early stopping patience’ from the set {50, 70} were optimized. All hidden layers are equipped with ReLU activation and the final layer is with sigmoid or softmax functions. For CNN, the parameters of ‘the number of batch sizes’ from the set {8, 16, 32, 64}, ‘the learning rate’ from the set {0.00001, 0.0001, 0.001}, ‘the dropout rate’ from the set {0.1, 0.2, 0.3, 0.4} were used.

**2.6 Construction of Protein-Protein Interaction (PPI) network for significant genes**

The PPI network of significant genes was constructed through the STRING online database (<https://string-db.org/>) [21]. To improve the quality of PPI network, we used the Cytoscape software [22]. The PPI network provides several nodes and edges, which indicate proteins and their interactions, respectively. The top-ranked genes were selected by using the topological analyses Degree method [23] of the PPI network. A node with the largest number of significant interactions/connections/edges with other nodes is considered as the top-ranked hub genes (HubGs).

We utilized the Human gene database (GeneCards) [24] and GeneMANIA [25] to validate the biological functions of the chosen genes. GeneCards aims to precisely identify enhancer elements and establish their links to genes, aiding in the comprehension of gene regulation and molecular pathways. Conversely, GeneMANIA identifies additional genes associated with a specific gene or a set of input genes through a comprehensive array of functional association data, such as protein and genetic interactions, pathways, co-expression, co-localization, and protein domain similarity.

**2.7 Regulatory network analysis of the top-ranked genes**

To explore key transcriptional regulatory transcription factors (TFs) of HubGs, we performed TFs-HubGs interaction network analysis by using the NetworkAnalyst web platform [26]. The TFs– HubGs interaction networks were constructed by using the JASPAR databases [27]. The Cytoscape software was used to construct the networks [22].

**2.8 Functional enrichment analysis**

Gene ontology (GO) functional and Kyoto Encyclopedia of Genes and Genomes (KEGG) pathway enrichment analysis [28,29] is a widely used approach to determine the significantly enriched functions and pathways by the identified HubGs. It is an important part for revealing the molecular mechanisms of actions and cellular roles of genes. The GO terms are categorized into Biological Process, Cellular Component, and Molecular Function [30]. We performed GO and KEGG enrichment analysis using Enrichr web tool (<https://maayanlab.cloud/Enrichr/>) [31]. The significance level was set to p-value < 0.05.

**2.9 Drug screening by molecular docking Study**

The molecular docking simulation study was adopted to perform the interaction among the target receptors and the drug molecules. Therefore, we collected the Food and Drug Administration (FDA) approved 47 drugs from DrugBank database (DrugBank[32] v6.0, https://go.drugbank.com), 215 FDA approved drugs from Drug-Gene Interaction Database (DGIdb[33] v4.2.0, https://www.dgidb.org/downloads), 1614 FDA approved drugs from ZINC database (<https://zinc.docking.org/substances/subsets/fda/>) and 210 drugs from literature review according to explore effective drugs for sarcopenia (**see Table S12**). Then, all drugs were compiled in Excel, where similar drugs were excluded, resulting in a list of 1940 unique drugs for analysis. Then, we obtained the three-dimensional structure of 1940 drugs from those literatures/databases.

In this study, docking analysis was performed between 7 drug target proteins and 1940 drugs (Table S7). The three dimensional (3D) structures of receptors were downloaded from Protein Data Bank (PDB) [34] and SWISS-MODEL [35]. The “Discovery Studio Visualizer” was used to visualize the 3D structures of protein interfaces [36]. PDB2PQR and H++ servers were utilized to assign the protonation state of target proteins [37,38]. All the missing hydrogen atoms were also appropriately added. The pKa for target proteins residues were investigated under the physical conditions of salinity = 0.15, internal dielectric = 10, pH = 7, and external dielectric = 80. On the other hand, the drugs were minimized energy through the Avogadro [39]. The target proteins were solvated with water, and only polar hydrogens were added. The receptor grid boxes (in X, Y, Z dimension) were prepared in the ADT4.2, and the pdbqt files of proteins were generated [40]. Similarly, the drug agents were prepared with default parameters, and only Gasteiger charges were added. Subsequently, molecular docking between receptors and drug agents were performed to calculate their binding affinities (kcal/mol) by using AutoDock Vina [41].

Flexible Ligand docking was performed applying the Lamarckian Genetic Algorithm with an exhaustiveness value of eight. The contributions of intramolecular hydrogen bonds, hydrophobic, ionic, and Van der Waals interactions between docked protein and ligand complexes were used to determine the free energy (ΔG) specifying affinity scoring of the binding. The docking poses were narrowed down using the force field’s free binding energy computation. After the docked protein-ligand complexes were created, the binding sites were analyzed to construct a 2D representation of the ligand interaction for each complex. The protein-ligand complexes were further visualized and analysis in Discovery Studio Visualizer [34] and PyMol [42]. Then, we selected the top-ranked drugs based on the docking score for further analysis.

Here, we calculated the inhibition constant (Ki) by converting the binding energy (ΔG) using the formula: Ki = exp(ΔG/RT), with R as the universal gas constant (1.985×10−3 kcal mol−1 K−1) and T as the temperature (298.15 K) [43].

**2.10 Docking validation process**

To validate the docking process, we performed a re-docking of the estradiol inhibitor with the ESR1 receptor using AutoDock-Vina. First, we manually removed the inhibitor from the original co-crystallized complex by editing the PDB file in a text editor. The inhibitor was then saved as a separate PDB file. We kept all the original grid parameters unchanged to ensure consistency in the docking process.

The purpose of this step was to confirm that the inhibitor would bind accurately to the active site with minimal deviation from the original co-crystallized structure. After re-docking, the new complex was superimposed onto the reference structure using PyMOL [42], and the root mean square deviation (RMSD) was calculated. The amino acid residues involved in binding were visualized and highlighted in a 2D image using Discovery Studio Visualizer [34]. This procedure was essential to validate the reliability of our docking process.

**Supplementary Results**

**3.1 Validate the biological functions of the seven genes using public databases**

We utilized the Human gene database (GeneCards) [24] and GeneMANIA [25] to validate the biological functions of the chosen genes. GeneCards aims to precisely identify enhancer elements and establish their links to genes, aiding in the comprehension of gene regulation and molecular pathways. Conversely, GeneMANIA identifies additional genes associated with a specific gene or a set of input genes through a comprehensive array of functional association data, such as protein and genetic interactions, pathways, co-expression, co-localization, and protein domain similarity. Our utilization of the GeneCards database offered insights into the regulatory elements (promoters and enhancers) for seven selected genes (*ESR1, ATM, CDC42, EP300, PIK3CA, EGF,* and *PTK2B*). **Table S9** displayed the Gene Name, GeneHancer (GH) ID, GH score, gene association (GA) score, total score and transcription binding sites, and the primary related disorders. According to this table, the chosen genes have outstanding GH score, GA score, total score, and literature reviews in the previous studies. Based on the disorder's description, the seven genes that were chosen have a strong correlation with complicated disorders, such as a higher chance of developing muscle-related diseases.

To demonstrate how effective these genes are, we also examined seven genes using a wide range of functional association data through GeneMANIA. **Figure S1** illustrated networks of genes related to *ESR1, ATM, CDC42, EP300, PIK3CA, EGF,* and *PTK2B*. This figure indicated that these chosen genes have robust connections through physical interactions, co-expression, predictions, co-localization, genetic interactions, pathways, and shared protein domains. Based on disease annotations, these 7 genes are closely linked to complex diseases, including a heightened risk of skeletal muscle cell-related disorders. Therefore, our model showed the ability to identify undiscovered genes from multi-omics datasets.

**3.2 Integration of transcription factor (TF) and gene expression**

Our previous experiments showed that our method can effectively predict factors related to sarcopenia using various biological datasets (multi-omics data). In this section, we explore whether our method performs well with an additional data type: networks of interactions between genes and TFs. We applied our method to predict ALM using the same parameters (described in the experimental settings section), focusing on TFs and their targets across three omics datasets. The performance metrics including RMSE, and MAE scores are detailed in **Table S10.** The RMSE of our model was 8.015, 8.144 and 8.212 for mRNA, meth, SNV omics datasets, respectively. On the other hand, the MAE of our model was and 6.320, 6.347, and 6.366 for mRNA, meth, SNV omics datasets, respectively. Our approach exhibited slightly lower performance in predicting lean mass for TFs-genes networks. However, when compared to the baseline methods, our approach demonstrated better performance. These findings signify that our proposed framework can work with varying sets of multi-omics data.

**3.3 Functional enrichment analysis of selected 16 genes**

The GO functional enrichment analysis of DEGs showed that 543 biological process (BP) terms, 52 cellular component (CC) terms and 84 molecular function (MF) terms are enriched by the selected 16 genes, where 7 PGs were involved with 323 BPs, 25 CCs and 48 MFs. On the other hand, 96 KEGG pathways are enriched by the selected 16 genes, where PGs were involved with 89 KEGG pathways (see **Figure 3 and Table S11**). The top five GO terms of the BPs, including 'Positive regulation of cell migration', 'Epidermal growth factor receptor signaling pathway', 'Apoptotic process', 'Cellular response to lipid', and 'Regulation of actin cytoskeleton organization' were significantly enriched by the PGs sets *{CDC42, EGF, PTK2B, ATM}, {PIK3CA, EGF, PTK2B}, {PIK3CA, EP300, PTK2B}, {PTK2B, ATM, ESR1},* and *{CDC42, PIK3CA, PTK2B},* respectively. The MFs GO terms 'Kinase binding', 'Protein kinase binding', 'Chromatin DNA binding', 'Transcription coregulator binding', and 'Nuclear receptor binding' were significantly enriched by the PGs sets *{CDC42, ESR1}, {CDC42, ESR1}, {EP300}, {EP300, ESR1},* and *{EP300, ESR1}*, respectively. The CCs GO terms 'Focal adhesion', 'Endocytic vesicle membrane', 'Clathrin-coated endocytic vesicle membrane', 'Clathrin-coated endocytic vesicle', and 'Intracellular membrane-bounded organelle' were significantly enriched by the SGBs sets *{CDC42, PTK2B}, {EGF}, {EGF}, {EGF},* and *{EP300, PTK2B, ATM, ESR1}*, respectively. We observed that KEGG pathway including 'HIF-1 signaling pathway', 'FoxO signaling pathway', 'Human papillomavirus infection', 'Shigellosis’, and ‘Salmonella infection' were significantly enriched by the PGs sets *{IL7R, CD33}, {EGF, IL7R}, {EGF, EP300, IL7R}, {EGF, PTK2B},* and *{EGF, EP300, IL7R},* respectively.

**3.4 Drug screening through docking study**

Molecular docking and virtual screening are speedy, cost-effective, and reliable methods for finding both a possible druggable target protein as well as a novel drug through computer-aided drug design (CADD) [44]. CADD is currently employed to swiftly annotate and assess vast pharmacological libraries [44].

This study applied molecular docking-based simulations to identify the promising drug agents for sarcopenia. Based on multi-omics integrated analysis, we discovered 7 human proteins which are involved in several biosynthetic pathways as a key regulator in sarcopenia. The probable molecular interactions of drug agents with these proteins were investigated in this *in-silico* study. We downloaded the 3D structure of our proposed 7 receptors (*ATM, EGF, EP300, CDC42,* *PTK2B, ESR1, PIK3CA*) from the Protein Data Bank (PDB) [34] with the PDB identifiers 5np0, 2kv4, 3i3j, 1a4r, 3cc6, 1a52, 2enq, respectively. Then, molecular docking was carried out between 7 receptors and 1940 drugs to calculate the binding affinity scores (kcal/mol) for each pair of receptors and agents. We used the binding energy in Kcal/mol to understand and explore how well different ligands or inhibitors bind to their respective protein targets. Basically, ligand's affinity for a receptor protein is generally higher the lower its binding energy. So, we sorted the target proteins by their binding affinity matrix's row sums of drug candidates across all target proteins in a descending order, and the drug agents according to column sums of target proteins across all drug candidates. The docking score for individual drugs against individual targets were recorded in **Table S13**.

**3.5 Identification of effective drugs for seven target genes**

According to AutoDock-vina software, the docking score is consistently presented as a negative value, where a more negative score indicates better potency (see **Table S13**). Then, we investigated the clinical development activities of selected drugs (see **Table 3**). Testosterone exhibited a docking score within −8.3 to −9.9 kcal/mol against the seven aging biomarkers. The highest potency was seen against *ESR1,* with a docking score of −9.9 kcal/mol, while the lowest was against *ATM,* with a docking score of −8.3 kcal/mol. Many researchers discovered that testosterone has a permissive effect on muscle mass and fiber cross-sectional area in parabiotic mice [45], increases muscle mass, and reduces fat mass [46], while at higher doses, it enhances both muscle and power [47]. Recent clinical studies have increasingly demonstrated the positive impact of testosterone on muscle function. For instance, in a phase III trial (NCT00799617; completed in 2014), testosterone treatment enhanced self-reported walking ability and the distance covered in the 6-minute walk test among elderly men with low testosterone levels, although it did not influence the rate of falls [48]. Similarly, in another phase II trial (NCT00104572; completed in 2015), this treatment led to improvements in fast gait speed at 3 and 12 months, as well as knee strength at 12 months, when compared to a placebo group [49]. Presently, clinical trials are increasingly examining the effects of testosterone in combination with other interventions, such as exercise (NCT02938923, recruiting). Mk0773 showed docking scores between -8.1 and -9.5 kcal/mol for the seven aging biomarkers. The most potent interaction was with *PTK2B* and *CDC42*, scoring -9.5 kcal/mol, while the weakest was with *ATM*, scoring -8.1 kcal/mol. MK-0773 is currently being studied in a clinical trial registered as NCT00529659. This trial is investigating the safety and effectiveness of MK-0773 in women with sarcopenia, a condition characterized by the loss of muscle mass [32]. The administration of MK‐0773 for a period of 6 months resulted in a notable increase in lean body mass, without any indication of androgenization. Nonetheless, there was no significant enhancement observed in muscle strength or physical performance [50]. Vorinostat demonstrated docking scores ranging from -7.3 to -9.2 kcal/mol for the seven aging biomarkers. The strongest interaction was observed with *PTK2B*, scoring -9.2 kcal/mol, whereas the least strong interaction was with *EGF*, scoring -8.1 kcal/mol. In investigation, it was found that vorinostat could enhance the functioning of mitochondria in skeletal muscles both *in-vitro* and *in-vivo* [51][52]. Galmozzi A. et al. explored how vorinostat (SAHA) boosted the expression of transcription factors and cofactors responsible for regulating mitochondrial function [51]. Vorinostat, a potent non-selective HDAC inhibitor, has received approval from the US Food and Drug Administration (FDA) for treating and managing cutaneous T-cell lymphoma [53]. Additionally, it was shown to influence cell cycle progression by restraining cell growth and encouraging differentiation [54].

On the other hand, canagliflozin demonstrated docking scores ranging from -7.8 to -9.5 kcal/mol against the seven aging biomarkers. The highest potency was observed against PTK2B, with a docking score of -9.9 kcal/mol, while the lowest was against PIK3CA, with a docking score of -8.3 kcal/mol. Canagliflozin, known as Invokana, is a medication used to manage type 2 diabetes mellitus in conjunction with lifestyle changes such as diet and exercise [55]. By promoting glucose excretion, canagliflozin can lead to weight loss, including a reduction in fat mass. A decrease in visceral fat may reduce inflammation and metabolic strain, factors that can adversely affect muscle health. Overall, our findings indicated that canagliflozin exhibited a comparatively similar potency to the other drugs.

**3.6 Analysis of the molecular binding pattern of canagliflozin with top-ranked target proteins**

Canagliflozin displayed binding energies of -9.5 kcal/mol, -8.7 kcal/mol, -9.0 kcal/mol, -8.3 kcal/mol, -8.5 kcal/mol, -8.1 kcal/mol, and -7.8 kcal/mol when interacting with *PTK2B, ESR1, CDC42, EP300, EGF, ATM, and PIK3CA* proteins, respectively (**Table 4**).

Interestingly in our *in-silico* analysis, we found that three key residues of active site viz., LYS457, GLU503, ASP567 of the human PTK2B protein interact with hydrogen bond with the three oxygen atoms of the canagliflozin. Thirteen other amino acid residues of the human *PTK2B* protein, LEU431, GLY432, GLY434, PHE435, PHE436, ALA455, VAL487, MET502, LEU504, ARG553, LEU556, PHE568, TYR573 formed Van-der-Waals interaction with canagliflozin (**Figure 4A (i-iii)**). The pi-alkyl interaction by VAL439, LEU570 with other unfavorable donor-donor interactions may help in stabilizing the canagliflozin bounded with the active residues of the human PTK2B protein (**Figure 4A (iii)**). This interaction showed the lowest binding energy of −9.5 kcal/mol and the highest inhibition constant of 6.97 µM, respectively. Based on these very similar binding patterns and docking complex analysis, we can say that canagliflozin is the true, potent inhibitor of the human *PTK2B* protein, thus it possibly helps in controlling sarcopenia. Canagliflozin also showed strong interaction with other six proteins based on the findings. Therefore, our *in-silico* findings indicate that canagliflozin could be a promising option for addressing sarcopenia.

**3.7 Re-docking and superimposition**

The re-docking was done to examine the docking procedure and efficiencies. The same methodology that was used previously was used in the re-docking process. The inhibitor bound exactly to the active site with a good binding energy of -10.1 kcal/mol.

The re-docked complex was then superimposed on to the native co-crystallized estradiol- ESR1 from PDB using PyMOL and a low RMSD of 0.0001 Å was observed (Figure S3). The re-docked complex was then superimposed onto the native co-crystallized estradiol- ESR1 using Discovery Studio Visualizer interestingly re-docked complex was superimposed completely onto the native co-crystallized complex without any adjustments. All the atoms of amino acids of both the complexes were superimposed without any constraints. On the whole, there were a total of 13 amino acid residues superimposed. The superimposed 2-dimensional structure and interacting amino acids are shown in **Table S15.**

**Supplemental Figure Titles and Legends**


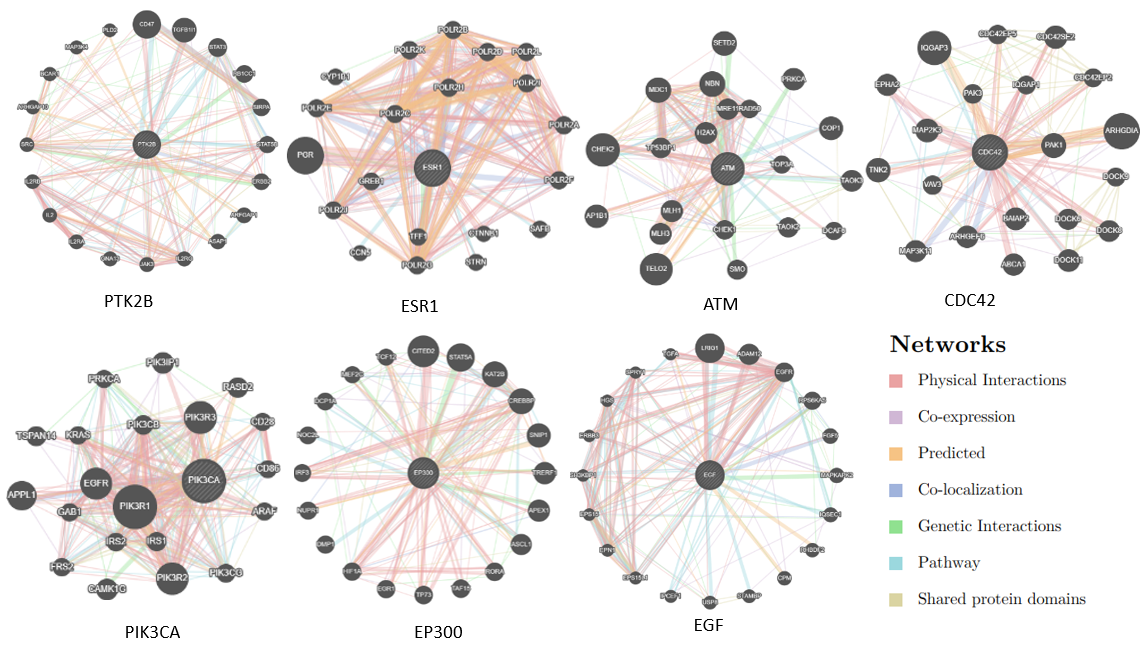


**Figure S1.** The network of predicted related genes of 7 significant genes enriched in GenAge Human Genes database.


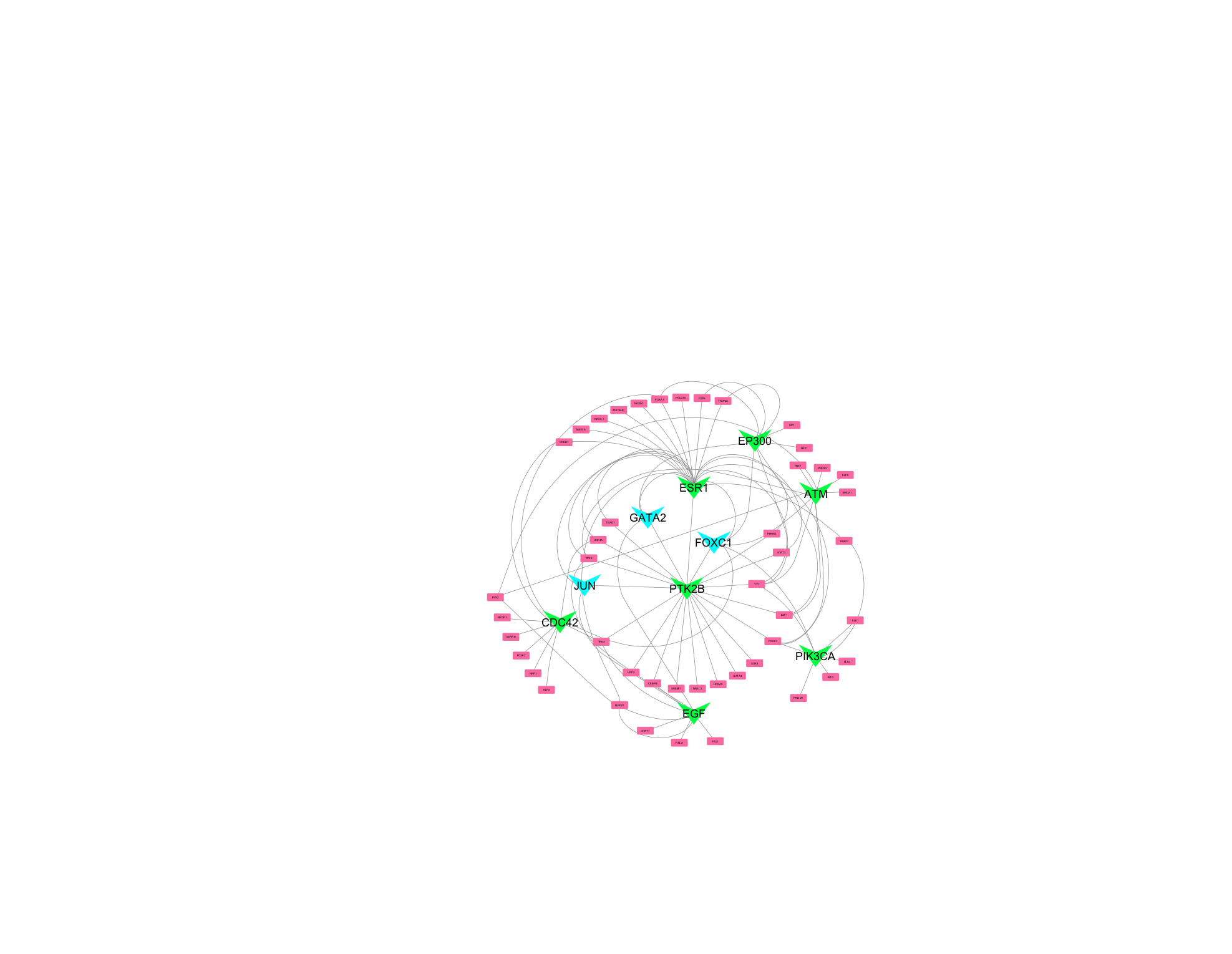


**Figure S2.** TFs-genes interaction network with selected 7 genes. The highlighted green-color nodes represent the selected 7 genes, cyan-color nodes represent the selected TFs (*GATA2, JUN, FOXC1*), and other pink-color nodes represent not selected TFs. The network consists of 50 nodes and 69 edges.


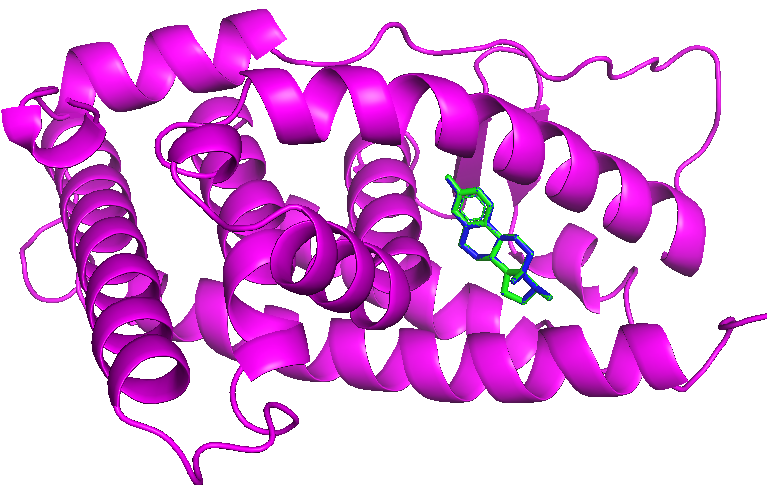


**Figure S3:** Superimposition of re-docked estradiol-ESR1 (Green) onto co-crystallized complex (Blue) in the active site using PyMOL (RMSD = 0.0001 Å)

**Supplemental Table Titles and Legends**

Table S1: Summary of omics data;

Table S2. The results of the grid search for CV1 and CV2 on biomedical multi-omics data lean mass prediction task; Table S3. The results of the grid search for CV3 and CV4 on biomedical multi-omics data lean mass prediction task;

Table S4. The results of the grid search for CV5 on biomedical multi-omics data lean mass prediction task;

Table S5: Comparison of the prediction performance of MTA-MO for two omics data integration;

Table S6: 639 significant genes selected by proposed method **(.xls);**

Table S7: Network analysis of each 16 significant genes using String database;

Table S8: The potential evidence and Cytogenetic information of 7 genes related to the human ageing process.

Table S9: The GeneHancer identifier, GeneHancer score, gene association score, total score, and major-related diseases of 7 selected genes

Table S10: Prediction performance of our method with Identified TFs;

Table S11: The top 20 significantly (p-value<0.05) enriched GO functions and KEGG pathways by significant 16 genes involving 7 selected with sarcopenia;

Table S12: The Food and Drug Administration (FDA) approved drugs list **(.xls);**

Table S13: The drug-protein interaction score based on molecular docking analysis **(.xls).**

Table S14: Comparison of the prediction performance of MTA-MO with XGBoost for the potential genes

Table S15: The drug-protein interaction score based on molecular docking analysis

**Table S1:** Summary of omics data

| **Omics Dataset** | **Age** | **Race** | **Number of samples** | **Number of Features** |
| --- | --- | --- | --- | --- |
| **SNV** | 20-50 | African-American and White | 1010 | 23536 |
| **mRNA** | 20-50 | African-American and White | 944 | 19286 |
| **meth** | 20-50 | African-American and White | 985 | 23688 |

**Table S2.** The results of the grid search for CV1 and CV2 on biomedical multi-omics data lean mass prediction task.

| **Outer CV** | **Number of Module** | **Early**  **Stopping**  **Patience** | **Learning**  **Rate** | **Weight**  **Decay** | **RMSE**  **For mRNA** | **RMSE**  **For meth** | **RMSE**  **For SNV** |  | **Outer CV** | **Number of Module** | **Early**  **Stopping**  **Patience** | **Learning**  **Rate** | **Weight**  **Decay** | **RMSE**  **For mRNA** | **RMSE**  **For meth** | **RMSE**  **For SNV** |
| --- | --- | --- | --- | --- | --- | --- | --- | --- | --- | --- | --- | --- | --- | --- | --- | --- |
| 1 | 32 | 50 | 0.00005 | 0.0 | 7.18 | 7.08 | 6.91 |  | 2 | 32 | 50 | 0.00005 | 0.0 | 7.22 | 7.15 | 7.28 |
| 1 | 32 | 50 | 0.00005 | 0.01 | 6.89 | 6.80 | 7.00 |  | 2 | 32 | 50 | 0.00005 | 0.01 | 7.80 | 7.61 | 8.31 |
| 1 | 32 | 50 | 0.00005 | 0.0001 | 7.73 | 7.78 | 7.95 |  | 2 | 32 | 50 | 0.00005 | 0.0001 | 8.01 | 8.00 | 8.08 |
| 1 | 32 | 50 | 0.000005 | 0.0 | 7.90 | 7.89 | 7.98 |  | 2 | 32 | 50 | 0.000005 | 0.0 | 8.05 | 8.08 | 8.15 |
| 1 | 32 | 50 | 0.000005 | 0.01 | 7.93 | 7.90 | 7.94 |  | 2 | 32 | 50 | 0.000005 | 0.01 | 8.05 | 8.08 | 8.14 |
| 1 | 32 | 50 | 0.000005 | 0.0001 | 7.92 | 7.89 | 7.93 |  | 2 | 32 | 50 | 0.000005 | 0.0001 | 8.06 | 8.10 | 8.14 |
| 1 | 32 | 50 | 0.0000005 | 0.0 | 7.92 | 7.89 | 7.92 |  | 2 | 32 | 50 | 0.0000005 | 0.0 | 8.04 | 8.08 | 8.15 |
| 1 | 32 | 50 | 0.0000005 | 0.01 | 7.92 | 7.89 | 7.92 |  | 2 | 32 | 50 | 0.0000005 | 0.01 | 8.04 | 8.08 | 8.14 |
| 1 | 32 | 50 | 0.0000005 | 0.0001 | 7.92 | 7.89 | 7.91 |  | 2 | 32 | 50 | 0.0000005 | 0.0001 | 8.04 | 8.08 | 8.13 |
| 1 | 32 | 100 | 0.00005 | 0.0 | 6.90 | 7.01 | 6.86 |  | 2 | 32 | 100 | 0.00005 | 0.0 | 8.25 | 8.00 | 7.43 |
| 1 | 32 | 100 | 0.00005 | 0.01 | 8.07 | 8.06 | 7.60 |  | 2 | 32 | 100 | 0.00005 | 0.01 | 7.95 | 8.49 | 9.38 |
| 1 | 32 | 100 | 0.00005 | 0.0001 | 8.29 | 8.20 | 8.17 |  | 2 | 32 | 100 | 0.00005 | 0.0001 | 7.91 | 7.92 | 8.15 |
| 1 | 32 | 100 | 0.000005 | 0.0 | 7.98 | 8.00 | 7.96 |  | 2 | 32 | 100 | 0.000005 | 0.0 | 7.97 | 8.04 | 8.27 |
| 1 | 32 | 100 | 0.000005 | 0.01 | 8.03 | 8.02 | 8.05 |  | 2 | 32 | 100 | 0.000005 | 0.01 | 8.04 | 8.08 | 8.25 |
| 1 | 32 | 100 | 0.000005 | 0.0001 | 8.02 | 8.02 | 8.02 |  | 2 | 32 | 100 | 0.000005 | 0.0001 | 8.06 | 8.08 | 8.22 |
| 1 | 32 | 100 | 0.0000005 | 0.0 | 8.02 | 8.03 | 8.03 |  | 2 | 32 | 100 | 0.0000005 | 0.0 | 8.05 | 8.08 | 8.20 |
| 1 | 32 | 100 | 0.0000005 | 0.01 | 8.04 | 8.04 | 8.05 |  | 2 | 32 | 100 | 0.0000005 | 0.01 | 8.05 | 8.08 | 8.18 |
| 1 | 32 | 100 | 0.0000005 | 0.0001 | 7.48 | 7.49 | 7.49 |  |  | 32 | 100 | 0.0000005 | 0.0001 | 7.39 | 7.63 | 7.46 |
| 1 | 64 | 50 | 0.00005 | 0.0 | 8.05 | 8.05 | 8.07 |  | 2 | 64 | 50 | 0.00005 | 0.0 | 8.05 | 8.08 | 8.17 |
| 1 | 64 | 50 | 0.00005 | 0.01 | 6.88 | 6.83 | 6.84 |  | 2 | 64 | 50 | 0.00005 | 0.01 | 7.22 | 7.15 | 7.18 |
| 1 | 64 | 50 | 0.00005 | 0.0001 | 7.82 | 7.84 | 8.65 |  | 2 | 64 | 50 | 0.00005 | 0.0001 | 7.29 | 7.29 | 7.28 |
| 1 | 64 | 50 | 0.000005 | 0.0 | 6.81 | 6.87 | 6.84 |  | 2 | 64 | 50 | 0.000005 | 0.0 | 7.97 | 7.95 | 8.10 |
| 1 | 64 | 50 | 0.000005 | 0.01 | 6.90 | 6.87 | 6.95 |  | 2 | 64 | 50 | 0.000005 | 0.01 | 8.04 | 7.97 | 8.12 |
| 1 | 64 | 50 | 0.000005 | 0.0001 | 6.94 | 6.91 | 6.81 |  | 2 | 64 | 50 | 0.000005 | 0.0001 | 8.08 | 8.00 | 8.14 |
| 1 | 64 | 50 | 0.0000005 | 0.0 | 6.88 | 6.86 | 7.00 |  | 2 | 64 | 50 | 0.0000005 | 0.0 | 8.09 | 8.03 | 8.15 |
| 1 | 64 | 50 | 0.0000005 | 0.01 | 6.89 | 6.87 | 6.97 |  | 2 | 64 | 50 | 0.0000005 | 0.01 | 8.10 | 8.03 | 8.15 |
| 1 | 64 | 50 | 0.0000005 | 0.0001 | 6.90 | 6.87 | 6.94 |  | 2 | 64 | 50 | 0.0000005 | 0.0001 | 8.10 | 8.05 | 8.15 |
| 1 | 64 | 100 | 0.00005 | 0.0 | 6.90 | 6.88 | 6.92 |  | 2 | 64 | 100 | 0.00005 | 0.0 | 8.10 | 8.04 | 8.14 |
| 1 | 64 | 100 | 0.00005 | 0.01 | 6.86 | 6.86 | 6.87 |  | 2 | 64 | 100 | 0.00005 | 0.01 | 7.16 | 7.15 | 7.17 |
| 1 | 64 | 100 | 0.00005 | 0.0001 | 7.26 | 6.94 | 7.27 |  | 2 | 64 | 100 | 0.00005 | 0.0001 | 8.12 | 7.74 | 7.73 |
| 1 | 64 | 100 | 0.000005 | 0.0 | 7.73 | 7.97 | 8.42 |  | 2 | 64 | 100 | 0.000005 | 0.0 | 7.33 | 8.03 | 7.76 |
| 1 | 64 | 100 | 0.000005 | 0.01 | 7.83 | 7.96 | 7.87 |  | 2 | 64 | 100 | 0.000005 | 0.01 | 7.95 | 7.89 | 7.91 |
| 1 | 64 | 100 | 0.000005 | 0.0001 | 7.87 | 7.97 | 7.91 |  | 2 | 64 | 100 | 0.000005 | 0.0001 | 7.97 | 7.87 | 7.91 |
| 1 | 64 | 100 | 0.0000005 | 0.0 | 7.90 | 7.99 | 7.94 |  | 2 | 64 | 100 | 0.0000005 | 0.0 | 7.98 | 7.86 | 7.91 |
| 1 | 64 | 100 | 0.0000005 | 0.01 | 7.92 | 8.01 | 7.96 |  | 2 | 64 | 100 | 0.0000005 | 0.01 | 7.98 | 7.86 | 7.91 |
| 1 | 64 | 100 | 0.0000005 | 0.0001 | 7.95 | 8.03 | 7.98 |  | 2 | 64 | 100 | 0.0000005 | 0.0001 | 7.98 | 7.87 | 7.91 |
| 1 | 128 | 50 | 0.00005 | 0.0 | 7.95 | 8.04 | 7.99 |  | 2 | 128 | 50 | 0.00005 | 0.0 | 7.99 | 7.87 | 7.92 |
| 1 | 128 | 50 | 0.00005 | 0.01 | 6.87 | 6.87 | 6.86 |  | 2 | 128 | 50 | 0.00005 | 0.01 | 7.18 | 7.13 | 7.13 |
| 1 | 128 | 50 | 0.00005 | 0.0001 | 7.89 | 9.32 | 9.93 |  | 2 | 128 | 50 | 0.00005 | 0.0001 | 7.40 | 8.30 | 8.00 |
| 1 | 128 | 50 | 0.000005 | 0.0 | 6.82 | 7.41 | 7.01 |  | 2 | 128 | 50 | 0.000005 | 0.0 | 7.80 | 7.98 | 7.85 |
| 1 | 128 | 50 | 0.000005 | 0.01 | 6.85 | 6.92 | 6.83 |  | 2 | 128 | 50 | 0.000005 | 0.01 | 8.06 | 7.96 | 7.82 |
| 1 | 128 | 50 | 0.000005 | 0.0001 | 6.94 | 6.82 | 7.14 |  | 2 | 128 | 50 | 0.000005 | 0.0001 | 8.02 | 7.94 | 7.83 |
| 1 | 128 | 50 | 0.0000005 | 0.0 | 6.79 | 6.87 | 6.82 |  | 2 | 128 | 50 | 0.0000005 | 0.0 | 8.00 | 7.92 | 7.83 |
| 1 | 128 | 50 | 0.0000005 | 0.01 | 6.80 | 6.87 | 6.83 |  | 2 | 128 | 50 | 0.0000005 | 0.01 | 7.99 | 7.92 | 7.83 |
| 1 | 128 | 50 | 0.0000005 | 0.0001 | 6.82 | 6.87 | 6.85 |  | 2 | 128 | 50 | 0.0000005 | 0.0001 | 7.99 | 7.91 | 7.84 |
| 1 | 128 | 100 | 0.00005 | 0.0 | 6.83 | 6.87 | 6.86 |  | 2 | 128 | 100 | 0.00005 | 0.0 | 7.98 | 7.91 | 7.84 |
| 1 | 128 | 100 | 0.00005 | 0.01 | 6.90 | 6.83 | 6.85 |  | 2 | 128 | 100 | 0.00005 | 0.01 | 7.18 | 7.17 | 7.18 |
| 1 | 128 | 100 | 0.00005 | 0.0001 | 7.34 | 6.89 | 7.88 |  | 2 | 128 | 100 | 0.00005 | 0.0001 | 7.50 | 7.20 | 8.42 |
| 1 | 128 | 100 | 0.000005 | 0.0 | 7.27 | 7.38 | 7.24 |  | 2 | 128 | 100 | 0.000005 | 0.0 | 8.37 | 8.57 | 8.58 |
| 1 | 128 | 100 | 0.000005 | 0.01 | 7.79 | 7.92 | 7.80 |  | 2 | 128 | 100 | 0.000005 | 0.01 | 7.99 | 7.97 | 7.94 |
| 1 | 128 | 100 | 0.000005 | 0.0001 | 7.79 | 7.90 | 7.83 |  | 2 | 128 | 100 | 0.000005 | 0.0001 | 7.99 | 7.94 | 7.92 |
| 1 | 128 | 100 | 0.0000005 | 0.0 | 7.80 | 7.90 | 7.85 |  | 2 | 128 | 100 | 0.0000005 | 0.0 | 7.98 | 7.93 | 7.91 |
| 1 | 128 | 100 | 0.0000005 | 0.01 | 7.80 | 7.90 | 7.86 |  | 2 | 128 | 100 | 0.0000005 | 0.01 | 7.97 | 7.92 | 7.90 |
| 1 | 128 | 100 | 0.0000005 | 0.0001 | 7.80 | 7.90 | 7.87 |  | 2 | 128 | 100 | 0.0000005 | 0.0001 | 7.96 | 7.91 | 7.89 |

**Table S3.** The results of the grid search for CV3 and CV4 on biomedical multi-omics data lean mass prediction task.

| **Outer CV** | **Number of Module** | **Early**  **Stopping**  **Patience** | **Learning**  **Rate** | **Weight**  **Decay** | **RMSE**  **For mRNA** | **RMSE**  **For meth** | **RMSE**  **For SNV** |  | **Outer CV** | **Number of Module** | **Early**  **Stopping**  **Patience** | **Learning**  **Rate** | **Weight**  **Decay** | **RMSE**  **For mRNA** | **RMSE**  **For meth** | **RMSE**  **For SNV** |
| --- | --- | --- | --- | --- | --- | --- | --- | --- | --- | --- | --- | --- | --- | --- | --- | --- |
| 3 | 32 | 50 | 0.00005 | 0.0 | 7.11 | 7.34 | 9.90 |  | 4 | 32 | 50 | 0.00005 | 0.0 | 7.38 | 7.32 | 7.60 |
| 3 | 32 | 50 | 0.00005 | 0.01 | 7.18 | 7.44 | 7.49 |  | 4 | 32 | 50 | 0.00005 | 0.01 | 7.57 | 7.67 | 7.92 |
| 3 | 32 | 50 | 0.00005 | 0.0001 | 6.91 | 6.98 | 7.00 |  | 4 | 32 | 50 | 0.00005 | 0.0001 | 8.51 | 8.57 | 8.77 |
| 3 | 32 | 50 | 0.000005 | 0.0 | 7.00 | 7.02 | 7.03 |  | 4 | 32 | 50 | 0.000005 | 0.0 | 8.41 | 8.49 | 8.64 |
| 3 | 32 | 50 | 0.000005 | 0.01 | 6.92 | 6.98 | 7.01 |  | 4 | 32 | 50 | 0.000005 | 0.01 | 8.43 | 8.52 | 8.64 |
| 3 | 32 | 50 | 0.000005 | 0.0001 | 7.00 | 7.03 | 7.03 |  | 4 | 32 | 50 | 0.000005 | 0.0001 | 8.43 | 8.53 | 8.62 |
| 3 | 32 | 50 | 0.0000005 | 0.0 | 6.99 | 7.02 | 7.03 |  | 4 | 32 | 50 | 0.0000005 | 0.0 | 8.43 | 8.53 | 8.62 |
| 3 | 32 | 50 | 0.0000005 | 0.01 | 6.98 | 7.01 | 7.03 |  | 4 | 32 | 50 | 0.0000005 | 0.01 | 8.43 | 8.54 | 8.61 |
| 3 | 32 | 50 | 0.0000005 | 0.0001 | 6.97 | 7.01 | 7.03 |  | 4 | 32 | 50 | 0.0000005 | 0.0001 | 8.43 | 8.54 | 8.61 |
| 3 | 32 | 100 | 0.00005 | 0.0 | 6.97 | 7.09 | 7.12 |  | 4 | 32 | 100 | 0.00005 | 0.0 | 8.23 | 8.35 | 7.53 |
| 3 | 32 | 100 | 0.00005 | 0.01 | 7.42 | 7.51 | 7.29 |  | 4 | 32 | 100 | 0.00005 | 0.01 | 7.94 | 7.48 | 7.71 |
| 3 | 32 | 100 | 0.00005 | 0.0001 | 7.11 | 7.25 | 7.42 |  | 4 | 32 | 100 | 0.00005 | 0.0001 | 8.01 | 8.31 | 8.97 |
| 3 | 32 | 100 | 0.000005 | 0.0 | 7.17 | 7.28 | 7.36 |  | 4 | 32 | 100 | 0.000005 | 0.0 | 8.06 | 8.28 | 8.38 |
| 3 | 32 | 100 | 0.000005 | 0.01 | 7.16 | 7.28 | 7.36 |  | 4 | 32 | 100 | 0.000005 | 0.01 | 8.06 | 8.32 | 8.33 |
| 3 | 32 | 100 | 0.000005 | 0.0001 | 7.16 | 7.30 | 7.35 |  | 4 | 32 | 100 | 0.000005 | 0.0001 | 8.07 | 8.34 | 8.32 |
| 3 | 32 | 100 | 0.0000005 | 0.0 | 7.17 | 7.30 | 7.35 |  | 4 | 32 | 100 | 0.0000005 | 0.0 | 8.08 | 8.35 | 8.31 |
| 3 | 32 | 100 | 0.0000005 | 0.01 | 7.18 | 7.31 | 7.34 |  | 4 | 32 | 100 | 0.0000005 | 0.01 | 8.08 | 8.34 | 8.31 |
| 3 | 32 | 100 | 0.0000005 | 0.0001 | 7.48 | 7.54 | 7.41 |  | 4 | 32 | 100 | 0.0000005 | 0.0001 | 7.38 | 7.39 | 7.51 |
| 3 | 64 | 50 | 0.00005 | 0.0 | 7.19 | 7.31 | 7.34 |  | 4 | 64 | 50 | 0.00005 | 0.0 | 8.08 | 8.35 | 8.29 |
| 3 | 64 | 50 | 0.00005 | 0.01 | 7.17 | 7.06 | 7.07 |  | 4 | 64 | 50 | 0.00005 | 0.01 | 7.39 | 7.37 | 7.46 |
| 3 | 64 | 50 | 0.00005 | 0.0001 | 7.22 | 6.96 | 7.39 |  | 4 | 64 | 50 | 0.00005 | 0.0001 | 7.54 | 7.77 | 7.94 |
| 3 | 64 | 50 | 0.000005 | 0.0 | 6.98 | 7.12 | 6.99 |  | 4 | 64 | 50 | 0.000005 | 0.0 | 7.52 | 7.35 | 7.60 |
| 3 | 64 | 50 | 0.000005 | 0.01 | 6.96 | 6.98 | 7.01 |  | 4 | 64 | 50 | 0.000005 | 0.01 | 7.34 | 7.33 | 7.40 |
| 3 | 64 | 50 | 0.000005 | 0.0001 | 7.00 | 7.01 | 7.05 |  | 4 | 64 | 50 | 0.000005 | 0.0001 | 7.47 | 7.55 | 7.39 |
| 3 | 64 | 50 | 0.0000005 | 0.0 | 6.95 | 6.99 | 6.98 |  | 4 | 64 | 50 | 0.0000005 | 0.0 | 7.32 | 7.32 | 7.35 |
| 3 | 64 | 50 | 0.0000005 | 0.01 | 6.95 | 6.99 | 6.98 |  | 4 | 64 | 50 | 0.0000005 | 0.01 | 7.32 | 7.33 | 7.36 |
| 3 | 64 | 50 | 0.0000005 | 0.0001 | 6.95 | 6.99 | 6.98 |  | 4 | 64 | 50 | 0.0000005 | 0.0001 | 7.33 | 7.34 | 7.36 |
| 3 | 64 | 100 | 0.00005 | 0.0 | 6.95 | 7.00 | 6.98 |  | 4 | 64 | 100 | 0.00005 | 0.0 | 7.34 | 7.35 | 7.37 |
| 3 | 64 | 100 | 0.00005 | 0.01 | 7.00 | 7.00 | 7.02 |  | 4 | 64 | 100 | 0.00005 | 0.01 | 7.36 | 7.37 | 7.36 |
| 3 | 64 | 100 | 0.00005 | 0.0001 | 6.99 | 6.93 | 6.96 |  | 4 | 64 | 100 | 0.00005 | 0.0001 | 8.21 | 7.99 | 7.67 |
| 3 | 64 | 100 | 0.000005 | 0.0 | 6.98 | 7.00 | 7.18 |  | 4 | 64 | 100 | 0.000005 | 0.0 | 7.85 | 8.06 | 8.03 |
| 3 | 64 | 100 | 0.000005 | 0.01 | 7.33 | 7.30 | 7.40 |  | 4 | 64 | 100 | 0.000005 | 0.01 | 8.15 | 8.19 | 8.25 |
| 3 | 64 | 100 | 0.000005 | 0.0001 | 7.34 | 7.30 | 7.40 |  | 4 | 64 | 100 | 0.000005 | 0.0001 | 8.20 | 8.21 | 8.27 |
| 3 | 64 | 100 | 0.0000005 | 0.0 | 7.35 | 7.31 | 7.40 |  | 4 | 64 | 100 | 0.0000005 | 0.0 | 8.21 | 8.21 | 8.27 |
| 3 | 64 | 100 | 0.0000005 | 0.01 | 7.35 | 7.30 | 7.39 |  | 4 | 64 | 100 | 0.0000005 | 0.01 | 8.21 | 8.22 | 8.27 |
| 3 | 64 | 100 | 0.0000005 | 0.0001 | 7.35 | 7.30 | 7.39 |  | 4 | 64 | 100 | 0.0000005 | 0.0001 | 8.22 | 8.22 | 8.28 |
| 3 | 128 | 50 | 0.00005 | 0.0 | 7.35 | 7.30 | 7.39 |  | 4 | 128 | 50 | 0.00005 | 0.0 | 8.22 | 8.23 | 8.28 |
| 3 | 128 | 50 | 0.00005 | 0.01 | 6.99 | 7.01 | 7.01 |  | 4 | 128 | 50 | 0.00005 | 0.01 | 7.39 | 7.38 | 7.39 |
| 3 | 128 | 50 | 0.00005 | 0.0001 | 7.43 | 7.77 | 7.57 |  | 4 | 128 | 50 | 0.00005 | 0.0001 | 8.11 | 8.22 | 8.37 |
| 3 | 128 | 50 | 0.000005 | 0.0 | 7.03 | 6.93 | 7.07 |  | 4 | 128 | 50 | 0.000005 | 0.0 | 7.28 | 7.28 | 7.30 |
| 3 | 128 | 50 | 0.000005 | 0.01 | 6.98 | 7.02 | 6.95 |  | 4 | 128 | 50 | 0.000005 | 0.01 | 7.36 | 7.49 | 7.47 |
| 3 | 128 | 50 | 0.000005 | 0.0001 | 7.00 | 6.94 | 7.24 |  | 4 | 128 | 50 | 0.000005 | 0.0001 | 7.31 | 7.28 | 7.30 |
| 3 | 128 | 50 | 0.0000005 | 0.0 | 6.97 | 6.97 | 6.96 |  | 4 | 128 | 50 | 0.0000005 | 0.0 | 7.40 | 7.48 | 7.48 |
| 3 | 128 | 50 | 0.0000005 | 0.01 | 6.97 | 6.97 | 6.95 |  | 4 | 128 | 50 | 0.0000005 | 0.01 | 7.39 | 7.44 | 7.45 |
| 3 | 128 | 50 | 0.0000005 | 0.0001 | 6.98 | 6.97 | 6.94 |  | 4 | 128 | 50 | 0.0000005 | 0.0001 | 7.38 | 7.41 | 7.42 |
| 3 | 128 | 100 | 0.00005 | 0.0 | 6.98 | 6.97 | 6.95 |  | 4 | 128 | 100 | 0.00005 | 0.0 | 7.37 | 7.38 | 7.40 |
| 3 | 128 | 100 | 0.00005 | 0.01 | 6.99 | 6.99 | 7.01 |  | 4 | 128 | 100 | 0.00005 | 0.01 | 7.36 | 7.38 | 7.37 |
| 3 | 128 | 100 | 0.00005 | 0.0001 | 6.96 | 6.96 | 6.98 |  | 4 | 128 | 100 | 0.00005 | 0.0001 | 8.82 | 8.75 | 9.02 |
| 3 | 128 | 100 | 0.000005 | 0.0 | 7.00 | 6.96 | 7.03 |  | 4 | 128 | 100 | 0.000005 | 0.0 | 7.37 | 7.37 | 7.38 |
| 3 | 128 | 100 | 0.000005 | 0.01 | 6.95 | 7.07 | 7.03 |  | 4 | 128 | 100 | 0.000005 | 0.01 | 7.27 | 7.29 | 7.32 |
| 3 | 128 | 100 | 0.000005 | 0.0001 | 7.09 | 7.01 | 7.19 |  | 4 | 128 | 100 | 0.000005 | 0.0001 | 7.44 | 7.44 | 7.54 |
| 3 | 128 | 100 | 0.0000005 | 0.0 | 6.96 | 6.94 | 7.03 |  | 4 | 128 | 100 | 0.0000005 | 0.0 | 7.28 | 7.30 | 7.31 |
| 3 | 128 | 100 | 0.0000005 | 0.01 | 6.95 | 6.95 | 7.00 |  | 4 | 128 | 100 | 0.0000005 | 0.01 | 7.28 | 7.30 | 7.32 |
| 3 | 128 | 100 | 0.0000005 | 0.0001 | 6.95 | 6.95 | 6.99 |  | 4 | 128 | 100 | 0.0000005 | 0.0001 | 7.29 | 7.31 | 7.33 |

**Table S4.** The results of the grid search for CV5 on biomedical multi-omics data lean mass prediction task.

| **Outer CV** | **Number of Module** | **Early**  **Stopping**  **Patience** | **Learning**  **Rate** | **Weight**  **Decay** | **RMSE**  **For mRNA** | **RMSE**  **For meth** | **RMSE**  **For SNV** |  | **Number of Module** | **Early**  **Stopping**  **Patience** | **Learning**  **Rate** | **Weight**  **Decay** | **RMSE**  **For mRNA** | **RMSE**  **For meth** | **RMSE**  **For SNV** |
| --- | --- | --- | --- | --- | --- | --- | --- | --- | --- | --- | --- | --- | --- | --- | --- |
|  |  |  |  |  |  |  |  |  | Average RMSE | | | | | | |
| 5 | 32 | 50 | 0.00005 | 0.0 | 7.29 | 7.12 | 7.22 |  | 32 | 50 | 0.00005 | 0.0 | 7.24 | 7.20 | 7.78 |
| 5 | 32 | 50 | 0.00005 | 0.01 | 7.65 | 7.90 | 7.73 |  | 32 | 50 | 0.00005 | 0.01 | 7.42 | 7.49 | 7.69 |
| 5 | 32 | 50 | 0.00005 | 0.0001 | 8.05 | 7.96 | 7.91 |  | 32 | 50 | 0.00005 | 0.0001 | 7.84 | 7.86 | 7.94 |
| 5 | 32 | 50 | 0.000005 | 0.0 | 8.01 | 7.95 | 7.89 |  | 32 | 50 | 0.000005 | 0.0 | 7.87 | 7.88 | 7.94 |
| 5 | 32 | 50 | 0.000005 | 0.01 | 7.99 | 7.95 | 7.87 |  | 32 | 50 | 0.000005 | 0.01 | 7.86 | 7.89 | 7.92 |
| 5 | 32 | 50 | 0.000005 | 0.0001 | 7.98 | 7.95 | 7.86 |  | 32 | 50 | 0.000005 | 0.0001 | 7.88 | 7.90 | 7.92 |
| 5 | 32 | 50 | 0.0000005 | 0.0 | 7.98 | 7.95 | 7.86 |  | 32 | 50 | 0.0000005 | 0.0 | 7.87 | 7.89 | 7.92 |
| 5 | 32 | 50 | 0.0000005 | 0.01 | 7.97 | 7.95 | 7.86 |  | 32 | 50 | 0.0000005 | 0.01 | 7.87 | 7.89 | 7.91 |
| 5 | 32 | 50 | 0.0000005 | 0.0001 | 7.96 | 7.94 | 7.86 |  | 32 | 50 | 0.0000005 | 0.0001 | 7.86 | 7.89 | 7.91 |
| 5 | 32 | 100 | 0.00005 | 0.0 | 8.81 | 7.16 | 7.64 |  | 32 | 100 | 0.00005 | 0.0 | 7.83 | 7.52 | 7.32 |
| 5 | 32 | 100 | 0.00005 | 0.01 | 7.37 | 7.43 | 7.44 |  | 32 | 100 | 0.00005 | 0.01 | 7.75 | 7.60 | 7.49 |
| 5 | 32 | 100 | 0.00005 | 0.0001 | 7.38 | 7.59 | 7.41 |  | 32 | 100 | 0.00005 | 0.0001 | 7.74 | 7.85 | 8.02 |
| 5 | 32 | 100 | 0.000005 | 0.0 | 7.28 | 7.41 | 7.32 |  | 32 | 100 | 0.000005 | 0.0 | 7.69 | 7.60 | 7.86 |
| 5 | 32 | 100 | 0.000005 | 0.01 | 7.29 | 7.42 | 7.34 |  | 32 | 100 | 0.000005 | 0.01 | 7.52 | 7.63 | 7.87 |
| 5 | 32 | 100 | 0.000005 | 0.0001 | 7.30 | 7.44 | 7.38 |  | 32 | 100 | 0.000005 | 0.0001 | 7.52 | 7.64 | 7.86 |
| 5 | 32 | 100 | 0.0000005 | 0.0 | 7.30 | 7.43 | 7.38 |  | 32 | 100 | 0.0000005 | 0.0 | 7.52 | 7.64 | 7.85 |
| 5 | 32 | 100 | 0.0000005 | 0.01 | 7.29 | 7.43 | 7.38 |  | 32 | 100 | 0.0000005 | 0.01 | 7.53 | 7.64 | 7.85 |
|  | 32 | 100 | 0.0000005 | 0.0001 | 7.38 | 7.59 | 7.41 |  | 32 | 100 | 0.0000005 | 0.0001 | 7.38 | 7.59 | 7.41 |
| 5 | 64 | 50 | 0.00005 | 0.0 | 7.29 | 7.43 | 7.38 |  | 64 | 50 | 0.00005 | 0.0 | 7.53 | 7.64 | 7.85 |
| 5 | 64 | 50 | 0.00005 | 0.01 | 7.15 | 7.11 | 7.16 |  | 64 | 50 | 0.00005 | 0.01 | 7.16 | 7.10 | 7.14 |
| 5 | 64 | 50 | 0.00005 | 0.0001 | 8.55 | 7.90 | 7.69 |  | 64 | 50 | 0.00005 | 0.0001 | 7.68 | 7.55 | 7.79 |
| 5 | 64 | 50 | 0.000005 | 0.0 | 7.58 | 7.50 | 7.48 |  | 64 | 50 | 0.000005 | 0.0 | 7.37 | 7.36 | 7.40 |
| 5 | 64 | 50 | 0.000005 | 0.01 | 7.71 | 7.51 | 7.54 |  | 64 | 50 | 0.000005 | 0.01 | 7.39 | 7.33 | 7.40 |
| 5 | 64 | 50 | 0.000005 | 0.0001 | 7.68 | 7.57 | 7.57 |  | 64 | 50 | 0.000005 | 0.0001 | 7.43 | 7.41 | 7.39 |
| 5 | 64 | 50 | 0.0000005 | 0.0 | 7.71 | 7.64 | 7.55 |  | 64 | 50 | 0.0000005 | 0.0 | 7.39 | 7.37 | 7.41 |
| 5 | 64 | 50 | 0.0000005 | 0.01 | 7.68 | 7.60 | 7.54 |  | 64 | 50 | 0.0000005 | 0.01 | 7.39 | 7.36 | 7.40 |
| 5 | 64 | 50 | 0.0000005 | 0.0001 | 7.66 | 7.57 | 7.52 |  | 64 | 50 | 0.0000005 | 0.0001 | 7.39 | 7.36 | 7.39 |
| 5 | 64 | 100 | 0.00005 | 0.0 | 7.64 | 7.55 | 7.52 |  | 64 | 100 | 0.00005 | 0.0 | 7.39 | 7.36 | 7.39 |
| 5 | 64 | 100 | 0.00005 | 0.01 | 7.10 | 7.13 | 7.16 |  | 64 | 100 | 0.00005 | 0.01 | 7.10 | 7.10 | 7.12 |
| 5 | 64 | 100 | 0.00005 | 0.0001 | 7.31 | 7.57 | 8.17 |  | 64 | 100 | 0.00005 | 0.0001 | 7.38 | 7.44 | 7.56 |
| 5 | 64 | 100 | 0.000005 | 0.0 | 7.31 | 7.37 | 7.63 |  | 64 | 100 | 0.000005 | 0.0 | 7.44 | 7.49 | 7.80 |
| 5 | 64 | 100 | 0.000005 | 0.01 | 7.92 | 8.01 | 8.03 |  | 64 | 100 | 0.000005 | 0.01 | 7.84 | 7.87 | 7.89 |
| 5 | 64 | 100 | 0.000005 | 0.0001 | 7.96 | 8.05 | 8.02 |  | 64 | 100 | 0.000005 | 0.0001 | 7.87 | 7.88 | 7.90 |
| 5 | 64 | 100 | 0.0000005 | 0.0 | 7.97 | 8.06 | 8.01 |  | 64 | 100 | 0.0000005 | 0.0 | 7.88 | 7.89 | 7.91 |
| 5 | 64 | 100 | 0.0000005 | 0.01 | 7.98 | 8.07 | 8.00 |  | 64 | 100 | 0.0000005 | 0.01 | 7.89 | 7.89 | 7.91 |
| 5 | 64 | 100 | 0.0000005 | 0.0001 | 7.99 | 8.07 | 8.00 |  | 64 | 100 | 0.0000005 | 0.0001 | 7.90 | 7.90 | 7.91 |
| 5 | 128 | 50 | 0.00005 | 0.0 | 7.99 | 8.07 | 8.00 |  | 128 | 50 | 0.00005 | 0.0 | 7.90 | 7.90 | 7.91 |
| 5 | 128 | 50 | 0.00005 | 0.01 | 7.15 | 7.08 | 7.11 |  | 128 | 50 | 0.00005 | 0.01 | 7.12 | 7.09 | 7.10 |
| 5 | 128 | 50 | 0.00005 | 0.0001 | 8.40 | 8.44 | 7.69 |  | 128 | 50 | 0.00005 | 0.0001 | 7.85 | 8.21 | 8.11 |
| 5 | 128 | 50 | 0.000005 | 0.0 | 7.39 | 7.30 | 7.48 |  | 128 | 50 | 0.000005 | 0.0 | 7.26 | 7.38 | 7.34 |
| 5 | 128 | 50 | 0.000005 | 0.01 | 7.42 | 7.27 | 7.47 |  | 128 | 50 | 0.000005 | 0.01 | 7.13 | 7.33 | 7.31 |
| 5 | 128 | 50 | 0.000005 | 0.0001 | 7.45 | 7.34 | 7.57 |  | 128 | 50 | 0.000005 | 0.0001 | 7.14 | 7.26 | 7.42 |
| 5 | 128 | 50 | 0.0000005 | 0.0 | 7.42 | 7.29 | 7.48 |  | 128 | 50 | 0.0000005 | 0.0 | 7.12 | 7.31 | 7.31 |
| 5 | 128 | 50 | 0.0000005 | 0.01 | 7.40 | 7.28 | 7.45 |  | 128 | 50 | 0.0000005 | 0.01 | 7.31 | 7.30 | 7.30 |
| 5 | 128 | 50 | 0.0000005 | 0.0001 | 7.38 | 7.27 | 7.43 |  | 128 | 50 | 0.0000005 | 0.0001 | 7.31 | 7.29 | 7.30 |
| 5 | 128 | 100 | 0.00005 | 0.0 | 7.36 | 7.26 | 7.42 |  | 128 | 100 | 0.00005 | 0.0 | 7.30 | 7.28 | 7.29 |
| 5 | 128 | 100 | 0.00005 | 0.01 | 7.17 | 7.14 | 7.10 |  | 128 | 100 | 0.00005 | 0.01 | 7.12 | 7.10 | 7.10 |
| 5 | 128 | 100 | 0.00005 | 0.0001 | 8.59 | 8.08 | 8.43 |  | 128 | 100 | 0.00005 | 0.0001 | 7.44 | 7.18 | 7.55 |
| 5 | 128 | 100 | 0.000005 | 0.0 | 7.28 | 7.24 | 7.25 |  | 128 | 100 | 0.000005 | 0.0 | 7.26 | 7.30 | 7.30 |
| 5 | 128 | 100 | 0.000005 | 0.01 | 7.29 | 7.25 | 7.27 |  | 128 | 100 | 0.000005 | 0.01 | 7.46 | 7.50 | 7.47 |
| 5 | 128 | 100 | 0.000005 | 0.0001 | 7.37 | 7.26 | 7.27 |  | 128 | 100 | 0.000005 | 0.0001 | 7.54 | 7.51 | 7.55 |
| 5 | 128 | 100 | 0.0000005 | 0.0 | 7.33 | 7.30 | 7.27 |  | 128 | 100 | 0.0000005 | 0.0 | 7.47 | 7.47 | 7.47 |
| 5 | 128 | 100 | 0.0000005 | 0.01 | 7.31 | 7.28 | 7.27 |  | 128 | 100 | 0.0000005 | 0.01 | 7.46 | 7.47 | 7.47 |
| 5 | 128 | 100 | 0.0000005 | 0.0001 | 7.30 | 7.27 | 7.27 |  | 128 | 100 | 0.0000005 | 0.0001 | 7.46 | 7.47 | 7.47 |

**Table S5:** Comparison of the prediction performance of MTA-MO for two omics data integration

| **Integration mRNA-meth** | | |
| --- | --- | --- |
| **mRNA** | | |
|  | **RMSE** | **MAE** |
| **MTA-MO** | 7.935 | 6.287 |
| **meth** | | |
| **MTA-MO** | 7.663 | 6.253 |
| **Integration mRNA-SNV** | | |
| **mRNA** | | |
| **MTA-MO** | 7.734 | 6.265 |
| **SNV** | | |
| **MTA-MO** | 7.880 | 6.278 |
| **Integration meth-SNV** | | |
| **meth** | | |
| **MTA-MO** | 7.572 | 5.685 |
| **SNV** | | |
| **MTA-MO** | 7.863 | 5.970 |

**Table S7:** Network analysis of each 16 significant genes using string database.

| **Genes** | **Number of edges** | **Average node degree** | **Average local clustering coefficient** | **Expected number of edges** | **PPI enrichment p-value** |
| --- | --- | --- | --- | --- | --- |
| ATM | 55 | 10 | 1 | 21 | 6.34e-10 |
| UBA52 | 55 | 10 | 1 | 27 | 1.27e-06 |
| CLTC | 53 | 9.64 | 0.968 | 11 | 1.0e-16 |
| TLR4 | 47 | 8.55 | 0.918 | 13 | 2.38e-13 |
| ESR1 | 50 | 9.09 | 0.914 | 23 | 9.83e-07 |
| CDC42 | 31 | 5.64 | 0.757 | 12 | 5.23e-06 |
| SMARCA4 | 55 | 10 | 1 | 11 | 1.0e-16 |
| EP300 | 47 | 8.55 | 0.902 | 29 | 0.00167 |
| GAPDH | 35 | 6.36 | 0.862 | 19 | 0.000486 |
| PTPRC | 32 | 5.82 | 0.804 | 15 | 0.000123 |
| PTK2B | 40 | 7.27 | 0.891 | 14 | 8.86e-09 |
| POLR2B | 55 | 10 | 1 | 15 | 4e-15 |
| CCND1 | 53 | 9.64 | 0.962 | 20 | 1.45e-09 |
| EGF | 54 | 9.82 | 0.982 | 21 | 5.64e-10 |
| DHX9 | 53 | 9.64 | 0.968 | 15 | 1.41e-14 |
| PIK3CA | 54 | 9.82 | 0.982 | 23 | 1.72e-08 |

**Table S8:** The potential evidence and cytogenetic information of 7 genes related to the human ageing process.

| **Gene Name** | **Aliases** | **Description** | **Cytogenetic information** | | |
| --- | --- | --- | --- | --- | --- |
|  |  |  | **Cytogenetic band** | **Location** | **Orientation** |
| *ATM* | *TEL1; TELO1; ATA; ATDC; ATC; ATD* | ATM is involved diverse aspects of cellular physiology including DNA repair and cell cycle control. It appears to activate DNA repair pathways in response to DNA damage [56]. | 11q22-q23 | 108,222,832 bp to 108,369,099 bp | Plus strand |
| *PIK3CA* | *PI3K* | PIK3CA plays a role in activating signaling cascades involved in cell growth, survival, proliferation, motility, and morphology. In mice, suppression of the activity of the p110alpha isoform of PIK3CA preserved cardiac function and prevented many age-associated changes in the heart [57]. | 3q26.3 | 179,148,523 bp to 179,234,709 bp | Plus strand |
| *CDC42* | *G25K; CDC42Hs* | CDC42 is regulates numerous signalling pathways, including cell cycle progression. Its activity increases with age in various mouse tissues. In mice, gene targeting of ARHGAP1, a negative regulator of CDC42, results in elevated levels of CDC42. CDC42 activation promotes a premature cellular senescence phenotype dependent on TP53 [58]. | 1p36.1 | 22,052,627 bp to 22,092,943 bp | Plus strand |
| *ESR1* | *NR3A1; Era; ESR* | ESR1 is a transcription factor that mediates the actions of estrogen. Polymorphisms in the human ESR1 gene have been associated with cardiovascular disease [59]. ESR1 has also been found to be upregulated in Alzheimer's disease [60]. A role for ESR1 in human ageing is plausible though at present largely obscure. | 6q25.1 | 151,807,678 bp to 152,103,273 bp | Plus strand |
| *EP300* | *p300; KAT3B* | EP300 is a transcriptional coactivator that mediates many transcriptional events including DNA repair. It also acts as a histone acetyltransferase to regulate transcription through chromatin structural changes[61]. EP300 activity is attenuated in ageing mice[62]. | 22q13.2 | 41,092,610 bp to 41,180,077 bp | Plus strand |
| *PTK2B* | *CAKB; PYK2; RAFTK; PTK; CADTK; FAK2* | Involved in stress response and signal transduction, PTK2B is an important player in a variety of processes including the regulation of ion channels by calcium and MAPK signalling [63]. PTK2B could potentially be involved in downstream signalling cascades related to ageing. | 8p21.1 | 27,311,482 bp to 27,459,391 bp | Plus strand |
| *EGF* | *-* | EGF stimulates the growth of several epidermal and epithelial cell types. Expression of EGF in transgenic mice causes stunted growth [64] | 4q25 | 109,912,884 bp to 110,012,962 bp | Plus strand |

**Table S9:** The GeneHancer identifier, GeneHancer score, gene association score, total score, and major-related diseases of 7 selected genes

| **Gene ID** | **GeneHancer (GH) ID** | **GH Score** | **Gene Association (GA) Score** | **Total Score** | **Transcription Binding Sites** | **Related Disease** |
| --- | --- | --- | --- | --- | --- | --- |
| PTK2B | GH08J027308 | 1.9 | 250.70 | 481.27 | 231 | Osteoporosis, Marble Bone, Osteopetrosis, Prostate Cancer |
| ATM | GH11J108219 | 2.4 | 262.10 | 621.35 | 264 | Breast Cancer, Lymphoma, T-Cell Prolymphocytic Leukemia |
| CDC42 | GH01J022051 | 2.2 | 256.80 | 573.36 | 273 | Bone marrow, Skeletal Muscle, Kidney, Thyroid |
| ESR1 | GH06J151689 | 1.6 | 252.30 | 411.39 | 52 | Muscle Benign Neoplasm, Muscle Tissue, Osteoporosis, Carcinoma |
| PIK3CA | GH03J179146 | 1.8 | 250.70 | 452.44 | 220 | Megalencephaly-Capillary Malformation-Polymicrogyria Syndrome, Ovarian Cancer, Cowden Syndrome 5, Adenocarcinoma |
| EP300 | GH22J041090 | 1.8 | 312.10 | 552.94 | 223 | Skeletal Muscle, Colorectal Cancer, Brachmann-De Lange Syndrome, Holt-Oram Syndrome |
| EGF | GH04J109980 | 1.2 | 266.00 | 325.79 | 11 | Breast Cyst, Short Bowel Nos, Mal De Meleda, Proconvertin Deficiency |

**Table S10:** Prediction performance of our method for transcription factors (TFs) and potential genes interaction network

| **mRNA** | | |
| --- | --- | --- |
|  | **RMSE** | **MAE** |
| XGBoost | 9.799 | 7.009 |
| CNN | 12.351 | 8.798 |
| DNN | 15.521 | 12.287 |
| **MTA-MO** | **8.015** | **6.320** |
| **meth** | | |
| XGBoost | 8.998 | 6.919 |
| CNN | 10.998 | 7.993 |
| DNN | 14.325 | 11.467 |
| **MTA-MO** | **8.144** | **6.347** |
| **SNV** | | |
| XGBoost | 9.154 | 7.948 |
| CNN | 11.456 | 8.875 |
| DNN | 15.765 | 13.987 |
| **MTA-MO** | **8.212** | **6.366** |

**Table S11:** The top 20 significantly (p-value<0.05) enriched GO functions and KEGG pathways by significant 16 genes involving 7 selected with sarcopenia.

| Biological Process (BP) | | | |
| --- | --- | --- | --- |
| **Term** | **BP** | **P-Value** | **Genes** |
| GO:0030335 | Positive Regulation Of Cell Migration | 1.73E-06 | **CDC42**; PTPRC; **EGF; PTK2B; ATM** |
| GO:0007173 | Epidermal Growth Factor Receptor Signaling Pathway | 5.84E-06 | **PIK3CA; EGF; PTK2B** |
| GO:0006915 | Apoptotic Process | 2.69E-05 | **PIK3CA; EP300; PTK2B;** GAPDH |
| GO:0071396 | Cellular Response To Lipid | 2.69E-05 | **PTK2B; ATM; ESR1;** TLR4 |
| GO:0032956 | Regulation Of Actin Cytoskeleton Organization | 4.89E-05 | **CDC42; PIK3CA; PTK2B** |
| GO:0045944 | Positive Regulation Of Transcription By RNA Polymerase II | 5.59E-05 | DHX9; **EP300; ATM; ESR1;** TLR4; SMARCA4 |
| GO:0043923 | Positive Regulation By Host Of Viral Transcription | 7.15E-05 | **EP300**; SMARCA4 |
| GO:1904892 | Regulation Of Receptor Signaling Pathway Via STAT | 8.10E-05 | PTPRC; **EGF** |
| GO:0043410 | Positive Regulation Of MAPK Cascade | 8.89E-05 | PTPRC; **EGF; PTK2B**; TLR4 |
| GO:0045740 | Positive Regulation Of DNA Replication | 9.11E-05 | **EGF**; DHX9 |
| GO:0001819 | Positive Regulation Of Cytokine Production | 1.01E-04 | PTPRC; DHX9; TLR4; GAPDH |
| GO:0071902 | Positive Regulation Of Protein Serine/Threonine Kinase Activity | 1.03E-04 | CCND1; **EGF**; TLR4 |
| GO:0032727 | Positive Regulation Of Interferon-Alpha Production | 1.25E-04 | DHX9; TLR4 |
| GO:0032680 | Regulation Of Tumor Necrosis Factor Production | 1.32E-04 | PTPRC; DHX9; TLR4 |
| GO:0010592 | Positive Regulation Of Lamellipodium Assembly | 1.50E-04 | **CDC42; PIK3CA** |
| GO:0001934 | Positive Regulation Of Protein Phosphorylation | 1.89E-04 | PTPRC; CCND1; **EGF; PTK2B** |
| GO:0032647 | Regulation Of Interferon-Alpha Production | 1.93E-04 | DHX9; TLR4 |
| GO:0060765 | Regulation Of Androgen Receptor Signaling Pathway | 2.08E-04 | **EP300**; SMARCA4 |
| GO:0051092 | Positive Regulation Of NF-kappaB Transcription Factor Activity | 2.24E-04 | DHX9; **EP300**; TLR4 |
| GO:1901701 | Cellular Response To Oxygen-Containing Compound | 2.51E-04 | **PTK2B; ATM; ESR1**; TLR4 |
|  |  |  |  |
| **Molecular Function (MF)** | | | |
| **Term** | **MF** | **PValue** | **Genes** |
| GO:0019900 | Kinase Binding | 2.23E-05 | **CDC42**; PTPRC; CCND1; CLTC; **ESR1** |
| GO:0019901 | Protein Kinase Binding | 3.69E-05 | **CDC42**; PTPRC; CCND1; CLTC; **ESR1** |
| GO:0031490 | Chromatin DNA Binding | 3.71E-05 | DHX9; **EP300**; SMARCA4 |
| GO:0001221 | Transcription Coregulator Binding | 6.29E-05 | **EP300; ESR1**; SMARCA4 |
| GO:0016922 | Nuclear Receptor Binding | 9.82E-05 | **EP300; ESR1**; SMARCA4 |
| GO:0097718 | Disordered Domain Specific Binding | 1.50E-04 | CLTC; GAPDH |
| GO:0050681 | Nuclear Androgen Receptor Binding | 1.78E-04 | **EP300**; SMARCA4 |
| GO:0001223 | Transcription Coactivator Binding | 3.32E-04 | **EP300; ESR1** |
| GO:0061629 | RNA Polymerase II-specific DNA-binding Transcription Factor Binding | 7.34E-04 | DHX9; **EP300**; SMARCA4 |
| GO:0003725 | Double-Stranded RNA Binding | 0.001211888 | DHX9; CLTC |
| GO:0034458 | 3'-5' RNA Helicase Activity | 0.003993945 | DHX9 |
| GO:0034190 | Apolipoprotein Receptor Binding | 0.003993945 | **CDC42** |
| GO:0046934 | Phosphatidylinositol-4,5-Bisphosphate 3-Kinase Activity | 0.003993945 | **PIK3CA** |
| GO:0035005 | 1-Phosphatidylinositol-4-Phosphate 3-Kinase Activity | 0.004790942 | **PIK3CA** |
| GO:0019828 | Aspartic-Type Endopeptidase Inhibitor Activity | 0.004790942 | GAPDH |
| GO:0032051 | Clathrin Light Chain Binding | 0.004790942 | CLTC |
| GO:0004972 | NMDA Glutamate Receptor Activity | 0.005587342 | **PTK2B** |
| GO:0034212 | Peptide N-acetyltransferase Activity | 0.006383144 | **EP300** |
| GO:0052813 | Phosphatidylinositol Bisphosphate Kinase Activity | 0.006383144 | **PIK3CA** |
| GO:0001164 | RNA Polymerase I Core Promoter Sequence-Specific DNA Binding | 0.006383144 | SMARCA4 |
|  |  |  |  |
| Cellular Component (CC) | | | |
| **Term** | **CC** | **PValue** | **Genes** |
| GO:0005925 | Focal Adhesion | 2.09E-04 | **CDC42**; PTPRC; CLTC; **PTK2B** |
| GO:0030666 | Endocytic Vesicle Membrane | 2.56E-04 | **EGF**; CLTC; UBA52 |
| GO:0030669 | Clathrin-Coated Endocytic Vesicle Membrane | 0.001325396 | **EGF**; CLTC |
| GO:0045334 | Clathrin-Coated Endocytic Vesicle | 0.002060726 | **EGF**; CLTC |
| GO:0043231 | Intracellular Membrane-Bounded Organelle | 0.002173552 | CCND1; DHX9; POLR2B; **EP300; PTK2B; ATM; ESR1;** UBA52; GAPDH; SMARCA4 |
| GO:0030665 | Clathrin-Coated Vesicle Membrane | 0.002306445 | **EGF**; CLTC |
| GO:0005765 | Lysosomal Membrane | 0.002636227 | **EGF**; CLTC; UBA52 |
| GO:0010008 | Endosome Membrane | 0.002742484 | CLTC; UBA52; TLR4 |
| GO:0030659 | Cytoplasmic Vesicle Membrane | 0.003386793 | PTPRC; UBA52; TLR4 |
| GO:0098588 | Bounding Membrane Of Organelle | 0.003424613 | **CDC42**; PTPRC; UBA52; TLR4 |
| GO:0017146 | NMDA Selective Glutamate Receptor Complex | 0.005587342 | **PTK2B** |
| GO:0070578 | RISC-loading Complex | 0.005587342 | DHX9 |
| GO:0072686 | Mitotic Spindle | 0.005704509 | **CDC42**; CLTC |
| GO:0005764 | Lysosome | 0.00693743 | **EGF**; CLTC; UBA52 |
| GO:0140092 | bBAF Complex | 0.00717835 | SMARCA4 |
| GO:0097440 | Apical Dendrite | 0.00717835 | **PTK2B** |
| GO:0071564 | npBAF Complex | 0.00956039 | SMARCA4 |
| GO:0140288 | GBAF Complex | 0.010353213 | SMARCA4 |
| GO:1990498 | Mitotic Spindle Microtubule | 0.010353213 | CLTC |
| GO:0016442 | RISC Complex | 0.010353213 | DHX9 |
|  |  |  |  |
| KEGG Pathway | | | |
| **KEGG Pathway** | | **PValue** | **Genes** |
| HIF-1 signaling pathway | | 1.83E-08 | **PIK3CA; EGF; EP300**; TLR4; GAPDH |
| FoxO signaling pathway | | 4.60E-08 | **PIK3CA**; CCND1; **EGF; EP300; ATM** |
| Human papillomavirus infection | | 1.37E-07 | **CDC42; PIK3CA**; CCND1; **EGF; EP300; ATM** |
| Shigellosis | | 1.06E-06 | **CDC42; PIK3CA; ATM**; UBA52; TLR4 |
| Salmonella infection | | 1.12E-06 | **CDC42; PTPRC; PIK3CA**; TLR4; GAPDH |
| Pathways in cancer | | 2.17E-06 | **CDC42; PIK3CA**; CCND1; **EGF; EP300; ESR1** |
| Thyroid hormone signaling pathway | | 2.19E-06 | **PIK3CA**; CCND1; **EP300; ESR1** |
| Yersinia infection | | 3.60E-06 | **CDC42; PIK3CA; PTK2B**; TLR4 |
| Breast cancer | | 4.76E-06 | **PIK3CA**; CCND1; **EGF; ESR1** |
| Hepatitis B | | 7.00E-06 | **PIK3CA; EP300; PTK2B**; TLR4 |
| JAK-STAT signaling pathway | | 7.00E-06 | **PIK3CA**; CCND1; **EGF; EP300** |
| Endometrial cancer | | 1.26E-05 | **PIK3CA**; CCND1; **EGF** |
| Kaposi sarcoma-associated herpesvirus infection | | 1.40E-05 | **PIK3CA**; CCND1; **EP300**; UBA52 |
| Focal adhesion | | 1.64E-05 | **CDC42; PIK3CA**; CCND1; **EGF** |
| Viral carcinogenesis | | 1.70E-05 | **CDC42; PIK3CA**; CCND1; **EP300** |
| Human immunodeficiency virus 1 infection | | 2.02E-05 | **PIK3CA; PTK2B; ATM**; TLR4 |
| Renal cell carcinoma | | 2.13E-05 | **CDC42; PIK3CA; EP300** |
| Prolactin signaling pathway | | 2.23E-05 | **PIK3CA**; CCND1; **ESR1** |
| Human T-cell leukemia virus 1 infection | | 2.30E-05 | **PIK3CA**; CCND1; **EP300; ATM** |
| Melanoma | | 2.42E-05 | **PIK3CA**; CCND1; **EGF** |

**Table S14:** Comparison of the prediction performance of MTA-MO with XGBoost for the potential genes

| **mRNA** | |
| --- | --- |
|  | **RMSE** |
| XGBoost | 13.512 |
| **MTA-MO** | **12.017** |
| **meth** | |
| XGBoost | 12.891 |
| **MTA-MO** | **12.436** |
| **SNV** | |
| XGBoost | 12.772 |
| **MTA-MO** | **12.003** |

Table S15: The drug-protein interaction score based on molecular docking analysis

| Complex |  | Hydrogen Bond | Hydrophobic Interaction | Two-dimensional image |
| --- | --- | --- | --- | --- |
| Co-crystallized complex | Leu346, Ala350, Glu353, Leu384, Leu387, Met388, Leu391, Arg394, Phe404, Met421, Ile424, His524, Leu525 | Glu353, Arg394, His524 | PHE404, LEU346, MET388, MET421, ILE424, LEU384, LEU525, HIS524, ALA350, LEU387, LEU391 | 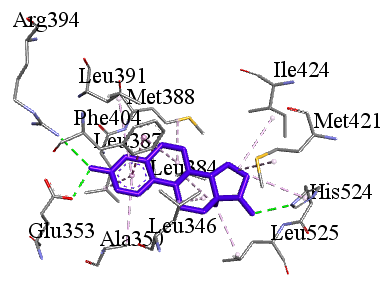 |
| Re-docked estradiol-ESR1 | Leu346, Ala350, Glu353, Leu384, Leu387, Met388, Leu391, Arg394, Phe404, Met421, Ile424, His524, Leu525 | Glu353, His524 | Phe404, Leu346, Met388, Met421, Ile424, Leu384, Leu525, His524, Ala350, Leu387, Leu391 | 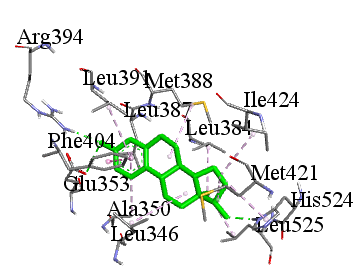 |

**Reference:**

1. Li, H.; Durbin, R. Fast and Accurate Short Read Alignment with Burrows-Wheeler Transform. *Bioinformatics* **2009**, doi:10.1093/bioinformatics/btp324.

2. McKenna, A.; Hanna, M.; Banks, E.; Sivachenko, A.; Cibulskis, K.; Kernytsky, A.; Garimella, K.; Altshuler, D.; Gabriel, S.; Daly, M.; et al. The Genome Analysis Toolkit: A MapReduce Framework for Analyzing next-Generation DNA Sequencing Data. *Genome Res.* **2010**, *20*, 1297–1303, doi:10.1101/gr.107524.110.

3. Van der Auwera, G.A.; Carneiro, M.O.; Hartl, C.; Poplin, R.; del Angel, G.; Levy-Moonshine, A.; Jordan, T.; Shakir, K.; Roazen, D.; Thibault, J.; et al. From FastQ Data to High-Confidence Variant Calls: The Genome Analysis Toolkit Best Practices Pipeline. *Curr. Protoc. Bioinforma.* **2013**, doi:10.1002/0471250953.bi1110s43.

4. Abyzov, A.; Urban, A.E.; Snyder, M.; Gerstein, M. CNVnator: An Approach to Discover, Genotype, and Characterize Typical and Atypical CNVs from Family and Population Genome Sequencing. *Genome Res.* **2011**, *21*, 974–984, doi:10.1101/gr.114876.110.

5. Chen, K.; Wallis, J.W.; McLellan, M.D.; Larson, D.E.; Kalicki, J.M.; Pohl, C.S.; McGrath, S.D.; Wendl, M.C.; Zhang, Q.; Locke, D.P.; et al. BreakDancer: An Algorithm for High-Resolution Mapping of Genomic Structural Variation. *Nat. Methods* **2009**, *6*, 677–681, doi:10.1038/nmeth.1363.

6. Fan, X.; Abbott, T.E.; Larson, D.; Chen, K. BreakDancer: Identification of Genomic Structural Variation from Paired-End Read Mapping. *Curr. Protoc. Bioinforma.* **2014**, doi:10.1002/0471250953.bi1506s45.

7. Wang, J.; Mullighan, C.G.; Easton, J.; Roberts, S.; Heatley, S.L.; Ma, J.; Rusch, M.C.; Chen, K.; Harris, C.C.; Ding, L.; et al. CREST Maps Somatic Structural Variation in Cancer Genomes with Base-Pair Resolution. *Nat. Methods* **2011**, *8*, 652–654, doi:10.1038/nmeth.1628.

8. Schulte-Sasse, R.; Budach, S.; Hnisz, D.; Marsico, A. Integration of Multiomics Data with Graph Convolutional Networks to Identify New Cancer Genes and Their Associated Molecular Mechanisms. *Nat. Mach. Intell.* **2021**, *3*, 513–526, doi:10.1038/s42256-021-00325-y.

9. Fujikawa, Y.; Sabokbar, A.; Neale, S.; Athanasou, N.A. Human Osteoclast Formation and Bone Resorption by Monocytes and Synovial Macrophages in Rheumatoid Arthritis. *Ann. Rheum. Dis.* **1996**, *55*, 816–822, doi:10.1136/ard.55.11.816.

10. Lari, R.; Kitchener, P.D.; Hamilton, J.A. The Proliferative Human Monocyte Subpopulation Contains Osteoclast Precursors. *Arthritis Res. Ther.* **2009**, *11*, doi:10.1186/ar2616.

11. Chatterjee, A.; Rodger, E.J.; Stockwell, P.A.; Weeks, R.J.; Morison, I.M. Technical Considerations for Reduced Representation Bisulfite Sequencing with Multiplexed Libraries. *J. Biomed. Biotechnol.* **2012**, *2012*, doi:10.1155/2012/741542.

12. Xi, Y.; Li, W. BSMAP: Whole Genome Bisulfite Sequence MAPping Program. *BMC Bioinformatics* **2009**, *10*, doi:10.1186/1471-2105-10-232.

13. Langmead, B.; Salzberg, S.L. Fast Gapped-Read Alignment with Bowtie 2. *Nat. Methods* **2012**, *9*, 357–359, doi:10.1038/nmeth.1923.

14. Kim, D.; Langmead, B.; Salzberg, S.L. HISAT: A Fast Spliced Aligner with Low Memory Requirements. *Nat. Methods* **2015**, *12*, 357–360, doi:10.1038/nmeth.3317.

15. Pertea, M.; Pertea, G.M.; Antonescu, C.M.; Chang, T.C.; Mendell, J.T.; Salzberg, S.L. StringTie Enables Improved Reconstruction of a Transcriptome from RNA-Seq Reads. *Nat. Biotechnol.* **2015**, *33*, 290–295, doi:10.1038/nbt.3122.

16. Robinson, M.D.; McCarthy, D.J.; Smyth, G.K. EdgeR: A Bioconductor Package for Differential Expression Analysis of Digital Gene Expression Data. *Bioinformatics* **2009**, *26*, doi:10.1093/bioinformatics/btp616.

17. Chu, T.; Wang, Z.; Pe’er, D.; Danko, C.G. Cell Type and Gene Expression Deconvolution with BayesPrism Enables Bayesian Integrative Analysis across Bulk and Single-Cell RNA Sequencing in Oncology. *Nat. Cancer* **2022**, *3*, 505–517, doi:10.1038/s43018-022-00356-3.

18. Hao, Y.; Stuart, T.; Kowalski, M.H.; Choudhary, S.; Hoffman, P.; Hartman, A.; Srivastava, A.; Molla, G.; Madad, S.; Fernandez-Granda, C.; et al. Dictionary Learning for Integrative, Multimodal and Scalable Single-Cell Analysis. *Nat. Biotechnol.* **2024**, *42*, 293–304, doi:10.1038/s41587-023-01767-y.

19. rajeshsharma7 Detect and Remove the Outliers Using Python Available online: https://www.geeksforgeeks.org/detect-and-remove-the-outliers-using-python/.

20. Kingma, D.P.; Ba, J.L. Adam: A Method for Stochastic Optimization. *3rd Int. Conf. Learn. Represent. ICLR 2015 - Conf. Track Proc.* **2015**.

21. Szklarczyk, D.; Franceschini, A.; Kuhn, M.; Simonovic, M.; Roth, A.; Minguez, P.; Doerks, T.; Stark, M.; Muller, J.; Bork, P.; et al. The STRING Database in 2011: Functional Interaction Networks of Proteins, Globally Integrated and Scored. *Nucleic Acids Res.* **2011**, *39*, doi:10.1093/nar/gkq973.

22. Shannon, P.; Markiel, A.; Ozier, O.; Baliga, N.S.; Wang, J.T.; Ramage, D.; Amin, N.; Schwikowski, B.; Ideker, T. Cytoscape: A Software Environment for Integrated Models of Biomolecular Interaction Networks. *Genome Res.* **2003**, *13*, doi:10.1101/gr.1239303.

23. Jeong, H.; Mason, S.P.; Barabási, A.L.; Oltvai, Z.N. Lethality and Centrality in Protein Networks. *Nature* **2001**, *411*, doi:10.1038/35075138.

24. Fishilevich, S.; Nudel, R.; Rappaport, N.; Hadar, R.; Plaschkes, I.; Stein, T.I.; Rosen, N.; Kohn, A.; Twik, M.; Safran, M.; et al. GeneHancer: Genome-Wide Integration of Enhancers and Target Genes in GeneCards. *Database* **2017**, *2017*, doi:10.1093/database/bax028.

25. Franz, M.; Rodriguez, H.; Lopes, C.; Zuberi, K.; Montojo, J.; Bader, G.D.; Morris, Q. GeneMANIA Update 2018. *Nucleic Acids Res.* **2018**, *46*, W60–W64, doi:10.1093/nar/gky311.

26. Zhou, G.; Soufan, O.; Ewald, J.; Hancock, R.E.W.; Basu, N.; Xia, J. NetworkAnalyst 3.0: A Visual Analytics Platform for Comprehensive Gene Expression Profiling and Meta-Analysis. *Nucleic Acids Res.* **2019**, *47*, doi:10.1093/nar/gkz240.

27. Rauluseviciute, I.; Riudavets-Puig, R.; Blanc-Mathieu, R.; Castro-Mondragon, J.A.; Ferenc, K.; Kumar, V.; Lemma, R.B.; Lucas, J.; Chèneby, J.; Baranasic, D.; et al. JASPAR 2024: 20th Anniversary of the Open-Access Database of Transcription Factor Binding Profiles. *Nucleic Acids Res.* **2023**, doi:10.1093/nar/gkad1059.

28. Boyle, E.I.; Weng, S.; Gollub, J.; Jin, H.; Botstein, D.; Cherry, J.M.; Sherlock, G. GO::TermFinder-Open Source Software for Accessing Gene Ontology Information and Finding Significantly Enriched Gene Ontology Terms Associated with a List of Genes INTRODUCTION: MOTIVATION AND DESIGN. *Bioinforma. Appl. NOTE* **2004**, *20*, 3710–3715, doi:10.1093/bioinformatics/bth456.

29. Kanehisa, M.; Goto, S. KEGG: Kyoto Encyclopedia of Genes and Genomes. *Nucleic Acids Res.* 2000, *28*.

30. Doms, A.; Schroeder, M. GoPubMed: Exploring PubMed with the Gene Ontology. *Nucleic Acids Res.* **2005**, *33*, doi:10.1093/nar/gki470.

31. Chen, E.Y.; Tan, C.M.; Kou, Y.; Duan, Q.; Wang, Z.; Meirelles, G. V.; Clark, N.R.; Ma’ayan, A. Enrichr: Interactive and Collaborative HTML5 Gene List Enrichment Analysis Tool. *BMC Bioinformatics* **2013**, *14*, doi:10.1186/1471-2105-14-128.

32. Knox, C.; Wilson, M.; Klinger, C.M.; Franklin, M.; Oler, E.; Wilson, A.; Pon, A.; Cox, J.; Chin, N.E.L.; Strawbridge, S.A.; et al. DrugBank 6.0: The DrugBank Knowledgebase for 2024. *Nucleic Acids Res.* **2024**, *52*, D1265–D1275, doi:10.1093/nar/gkad976.

33. Freshour, S.L.; Kiwala, S.; Cotto, K.C.; Coffman, A.C.; McMichael, J.F.; Song, J.J.; Griffith, M.; Griffith, O.L.; Wagner, A.H. Integration of the Drug-Gene Interaction Database (DGIdb 4.0) with Open Crowdsource Efforts. *Nucleic Acids Res.* **2021**, *49*, D1144–D1151, doi:10.1093/nar/gkaa1084.

34. Berman, H.M.; Westbrook, J.; Feng, Z.; Gilliland, G.; Bhat, T.N.; Weissig, H.; Shindyalov, I.N.; Bourne, P.E. The Protein Data Bank. *Nucleic Acids Res.* 2000.

35. Waterhouse, A.; Bertoni, M.; Bienert, S.; Studer, G.; Tauriello, G.; Gumienny, R.; Heer, F.T.; De Beer, T.A.P.; Rempfer, C.; Bordoli, L.; et al. SWISS-MODEL: Homology Modelling of Protein Structures and Complexes. *Nucleic Acids Res.* **2018**, doi:10.1093/nar/gky427.

36. Studio, D. Discovery Studio Visualizer. *Discovery* **2014**, 3–5.

37. Dolinsky, T.J.; Czodrowski, P.; Li, H.; Nielsen, J.E.; Jensen, J.H.; Klebe, G.; Baker, N.A. PDB2PQR: Expanding and Upgrading Automated Preparation of Biomolecular Structures for Molecular Simulations. *Nucleic Acids Res.* **2007**, *35*, doi:10.1093/nar/gkm276.

38. Gordon, J.C.; Myers, J.B.; Folta, T.; Shoja, V.; Heath, L.S.; Onufriev, A. H++: A Server for Estimating PKas and Adding Missing Hydrogens to Macromolecules. *Nucleic Acids Res.* **2005**, *33*, doi:10.1093/nar/gki464.

39. Hanwell, M.D.; Curtis, D.E.; Lonie, D.C.; Vandermeerschd, T.; Zurek, E.; Hutchison, G.R. Avogadro: An Advanced Semantic Chemical Editor, Visualization, and Analysis Platform. *J. Cheminform.* **2012**, *4*, doi:10.1186/1758-2946-4-17.

40. Morris, G.M.; Ruth, H.; Lindstrom, W.; Sanner, M.F.; Belew, R.K.; Goodsell, D.S.; Olson, A.J. Software News and Updates AutoDock4 and AutoDockTools4: Automated Docking with Selective Receptor Flexibility. *J. Comput. Chem.* **2009**, *30*, 2785–2791, doi:10.1002/jcc.21256.

41. Trott, O.; Olson, A.J. AutoDock Vina: Improving the Speed and Accuracy of Docking with a New Scoring Function, Efficient Optimization, and Multithreading. *J. Comput. Chem.* **2009**, NA-NA, doi:10.1002/jcc.21334.

42. Delano, W.L.; Bromberg, S. *PyMOL User’s Guide*; 2004;

43. Ortiz, C.L.D.; Completo, G.C.; Nacario, R.C.; Nellas, R.B. Potential Inhibitors of Galactofuranosyltransferase 2 (GlfT2): Molecular Docking, 3D-QSAR, and In Silico ADMETox Studies. *Sci. Rep.* **2019**, *9*, doi:10.1038/s41598-019-52764-8.

44. Ashraf, S.A.; Elkhalifa, A.E.O.; Mehmood, K.; Adnan, M.; Khan, M.A.; Eltoum, N.E.; Krishnan, A.; Baig, M.S. Multi-Targeted Molecular Docking, Pharmacokinetics, and Drug-Likeness Evaluation of Okra-Derived Ligand Abscisic Acid Targeting Signaling Proteins Involved in the Development of Diabetes. *Molecules* **2021**, *26*, doi:10.3390/molecules26195957.

45. Sinha, I.; Sinha-Hikim, A.P.; Wagers, A.J.; Sinha-Hikim, I. Testosterone Is Essential for Skeletal Muscle Growth in Aged Mice in a Heterochronic Parabiosis Model. *Cell Tissue Res.* **2014**, *357*, 815–821, doi:10.1007/s00441-014-1900-2.

46. Wittert, G.A.; Chapman, I.M.; Haren, M.T.; Mackintosh, S.; Coates, P.; Morley, J.E. Oral Testosterone Supplementation Increases Muscle and Decreases Fat Mass in Healthy Elderly Males with Low-Normal Gonadal Status. *Journals Gerontol. - Ser. A Biol. Sci. Med. Sci.* **2003**, *58*, 618–625, doi:10.1093/gerona/58.7.m618.

47. Bhasin, S.; Woodhouse, L.; Casaburi, R.; Singh, A.B.; Mac, R.P.; Lee, M.; Yarasheski, K.E.; Sinha-Hikim, I.; Dzekov, C.; Dzekov, J.; et al. Older Men Are as Responsive as Young Men to the Anabolic Effects of Graded Doses of Testosterone on the Skeletal Muscle. *J. Clin. Endocrinol. Metab.* **2005**, *90*, 678–688, doi:10.1210/jc.2004-1184.

48. Bhasin, S.; Ellenberg, S.S.; Storer, T.W.; Basaria, S.; Pahor, M.; Stephens-Shields, A.J.; Cauley, J.A.; Ensrud, K.E.; Farrar, J.T.; Cella, D.; et al. Effect of Testosterone Replacement on Measures of Mobility in Older Men with Mobility Limitation and Low Testosterone Concentrations: Secondary Analyses of the Testosterone Trials. *Lancet Diabetes Endocrinol.* **2018**, *6*, 879–890, doi:10.1016/S2213-8587(18)30171-2.

49. Dias, J.P.; Veldhuis, J.D.; Carlson, O.; Shardell, M.; Chia, C.W.; Melvin, D.; Egan, J.M.; Basaria, S. Effects of Transdermal Testosterone Gel or an Aromatase Inhibitor on Serum Concentration and Pulsatility of Growth Hormone in Older Men with Age-Related Low Testosterone. *Metabolism.* **2017**, *69*, 143–147, doi:10.1016/j.metabol.2017.01.025.

50. Papanicolaou, D.A.; Ather, S.N.; Zhu, H.; Zhou, Y.; Lutkiewicz, J.; Scott, B.B.; Chandler, J. A Phase IIA Randomized, Placebo-Controlled Clinical Trial to Study the Efficacy and Safety of the Selective Androgen Receptor Modulator (SARM), MK-0773 in Female Participants with Sarcopenia. *J. Nutr. Heal. Aging* **2013**, *17*, 533–543, doi:10.1007/s12603-013-0335-x.

51. Galmozzi, A.; Mitro, N.; Ferrari, A.; Gers, E.; Gilardi, F.; Godio, C.; Cermenati, G.; Gualerzi, A.; Donetti, E.; Rotili, D.; et al. Inhibition of Class i Histone Deacetylases Unveils a Mitochondrial Signature and Enhances Oxidative Metabolism in Skeletal Muscle and Adipose Tissue. *Diabetes* **2013**, *62*, 732–742, doi:10.2337/db12-0548.

52. Liang, S.; Liu, D.; Xiao, Z.; Greenbaum, J.; Shen, H.; Xiao, H.; Deng, H. Repurposing Approved Drugs for Sarcopenia Based on Transcriptomics Data in Humans. *Pharmaceuticals* **2023**, *16*, doi:10.3390/ph16040607.

53. Cole, J.J.; Robertson, N.A.; Rather, M.I.; Thomson, J.P.; McBryan, T.; Sproul, D.; Wang, T.; Brock, C.; Clark, W.; Ideker, T.; et al. Diverse Interventions That Extend Mouse Lifespan Suppress Shared Age-Associated Epigenetic Changes at Critical Gene Regulatory Regions. *Genome Biol.* **2017**, *18*, doi:10.1186/s13059-017-1185-3.

54. Richon, V.M.; Garcia-Vargas, J.; Hardwick, J.S. Development of Vorinostat: Current Applications and Future Perspectives for Cancer Therapy. *Cancer Lett.* **2009**, *280*, 201–210, doi:10.1016/j.canlet.2009.01.002.

55. Lamos, E.M.; Younk, L.M.; Davis, S.N. Canagliflozin, an Inhibitor of Sodium-Glucose Cotransporter 2, for the Treatment of Type 2 Diabetes Mellitus. *Expert Opin. Drug Metab. Toxicol.* **2013**, *9*, 763–775, doi:10.1517/17425255.2013.791282.

56. Rotman, G.; Shiloh, Y. Ataxia-Telangiectasia: Is ATM a Sensor of Oxidative Damage and Stress? *BioEssays* **1997**, *19*, 911–917, doi:10.1002/bies.950191011.

57. Inuzuka, Y.; Okuda, J.; Kawashima, T.; Kato, T.; Niizuma, S.; Tamaki, Y.; Iwanaga, Y.; Yoshida, Y.; Kosugi, R.; Watanabe-Maeda, K.; et al. Suppression of Phosphoinositide 3-Kinase Prevents Cardiac Aging in Mice. *Circulation* **2009**, *120*, 1695–1703, doi:10.1161/CIRCULATIONAHA.109.871137.

58. Wang, L.; Yang, L.; Debidda, M.; Witte, D.; Zheng, Y. Cdc42 GTPase-Activating Protein Deficiency Promotes Genomic Instability and Premature Aging-like Phenotypes. *Proc. Natl. Acad. Sci. U. S. A.* **2007**, *104*, 1248–1253, doi:10.1073/pnas.0609149104.

59. Shearman, A.M.; Cupples, L.A.; Demissie, S.; Peter, I.; Schmid, C.H.; Karas, R.H.; Mendelsohn, M.L.; Housman, D.E.; Levy, D. Association between Estrogen Receptor α Gene Variation and Cardiovascular Disease. *Jama* **2003**, *290*, 2263–2270, doi:10.1001/jama.290.17.2263.

60. Ishunina, T.A.; Swaab, D.F. Increased Expression of Estrogen Receptor α and β in the Nucleus Basalis of Meynert in Alzheimer’s Disease. *Neurobiol. Aging* **2001**, *22*, 417–426, doi:10.1016/S0197-4580(00)00255-4.

61. Hasan, S.; Hassa, P.O.; Imhof, R.; Hottiger, M.O. Transcription Coactivator P300 Binds PCNA and May Have a Role in DNA Repair Synthesis. *Nature* **2001**, *410*, 387–391, doi:10.1038/35066610.

62. Li, Q.; Xiao, H.; Isobe, K.I. Histone Acetyltransferase Activities of CAMP-Regulated Enhancer-Binding Protein and P300 in Tissues of Fetal, Young, and Old Mice. *Journals Gerontol. - Ser. A Biol. Sci. Med. Sci.* **2002**, *57*, doi:10.1093/gerona/57.3.B93.

63. Lev, S.; Moreno, H.; Martinez, R.; Canoll, P.; Peles, E.; Musacchio, J.M.; Plowman, G.D.; Rudy, B.; Schlessinger, J. Protein Tyrosine Kinase PYK2 Involved in Ca2+-Induced Regulation of Ion Channel and MAP Kinase Functions. *Nature* **1995**, *376*, 737–745, doi:10.1038/376737a0.

64. Chan, S.Y.; Wong, R.W.C. Expression of Epidermal Growth Factor in Transgenic Mice Causes Growth Retardation. *J. Biol. Chem.* **2000**, *275*, 38693–38698, doi:10.1074/jbc.M004189200.

**Supplementary Reference**

S1. Fleury, N.; Geldenhuys, S.; Gorman, S. Sun Exposure and Its Effects on Human Health: Mechanisms through Which Sun Exposure Could Reduce the Risk of Developing Obesity and Cardiometabolic Dysfunction. *Int. J. Environ. Res. Public Health* **2016**, *13*, doi:10.3390/ijerph13100999.

S2. Graff-Iversen, S.; Hewitt, S.; Forsén, L.; Grøtvedt, L.; Ariansen, I. Associations of Tobacco Smoking with Body Mass Distribution; A Population-Based Study of 65,875 Men and Women in Midlife. *BMC Public Health* **2019**, *19*, doi:10.1186/s12889-019-7807-9.

S3. Liangpunsakul, S.; Crabb, D.W.; Qi, R. Relationship among Alcohol Intake, Body Fat, and Physical Activity: A Population-Based Study. *Ann. Epidemiol.* **2010**, *20*, 670–675, doi:10.1016/j.annepidem.2010.05.014.

S4. Radavelli-Bagatini, S.; Zhu, K.; Lewis, J.R.; Dhaliwal, S.S.; Prince, R.L. Association of Dairy Intake with Body Composition and Physical Function in Older Community-Dwelling Women. *J. Acad. Nutr. Diet.* **2013**, *113*, 1669–1674, doi:10.1016/j.jand.2013.05.019.

S5. GenAge Human Genes Available online: https://genomics.senescence.info/genes/human.html (accessed on 8 January 2024).

S6. Knox, C.; Wilson, M.; Klinger, C.M.; Franklin, M.; Oler, E.; Wilson, A.; Pon, A.; Cox, J.; Chin, N.E.L.; Strawbridge, S.A.; et al. DrugBank 6.0: The DrugBank Knowledgebase for 2024. *Nucleic Acids Res.* **2024**, *52*, D1265–D1275, doi:10.1093/nar/gkad976.

S7. Freshour, S.L.; Kiwala, S.; Cotto, K.C.; Coffman, A.C.; McMichael, J.F.; Song, J.J.; Griffith, M.; Griffith, O.L.; Wagner, A.H. Integration of the Drug-Gene Interaction Database (DGIdb 4.0) with Open Crowdsource Efforts. *Nucleic Acids Res.* **2021**, *49*, D1144–D1151, doi:10.1093/nar/gkaa1084.

S8. Fishilevich, S.; Nudel, R.; Rappaport, N.; Hadar, R.; Plaschkes, I.; Stein, T.I.; Rosen, N.; Kohn, A.; Twik, M.; Safran, M.; et al. GeneHancer: Genome-Wide Integration of Enhancers and Target Genes in GeneCards. *Database* **2017**, *2017*, doi:10.1093/database/bax028.

S9. Franz, M.; Rodriguez, H.; Lopes, C.; Zuberi, K.; Montojo, J.; Bader, G.D.; Morris, Q. GeneMANIA Update 2018. *Nucleic Acids Res.* **2018**, *46*, W60–W64, doi:10.1093/nar/gky311.

S10. Matsubara, T.; Yaginuma, T.; Addison, W.N.; Fujita, Y.; Watanabe, K.; Yoshioka, I.; Hikiji, H.; Maki, K.; Baron, R.; Kokabu, S. Plectin Stabilizes Microtubules during Osteoclastic Bone Resorption by Acting as a Scaffold for Src and Pyk2. *Bone* **2020**, *132*, doi:10.1016/j.bone.2019.115209.

S11. Fan, L.; Lu, Y.; Shen, X.; Shao, H.; Suo, L.; Wu, Q. Alpha Protocadherins and Pyk2 Kinase Regulate Cortical Neuron Migration and Cytoskeletal Dynamics via Rac1 GTPase and WAVE Complex in Mice. *Elife* **2018**, *7*, doi:10.7554/eLife.35242.

S12. Li, X.; Huynh, H.; Zuo, H.; Salminen, M.; Wan, Y. Gata2 Is a Rheostat for Mesenchymal Stem Cell Fate in Male Mice. *Endocrinology* **2016**, *157*, 1021–1028, doi:10.1210/en.2015-1827.

S13. Puntschart, A.; Wey, E.; Jostarndt, K.; Vogt, M.; Wittwer, M.; Widmer, H.R.; Hoppeler, H.; Billeter, R. Expression of Fos and Jun Genes in Human Skeletal Muscle after Exercise. *Am. J. Physiol. - Cell Physiol.* **1998**, *274*, doi:10.1152/ajpcell.1998.274.1.c129.

S14. Wang, R.; Wang, W.Q.; Li, X.Q.; Zhao, J.; Yang, K.; Feng, Y.; Guo, M.M.; Liu, M.; Liu, X.; Wang, X.; et al. A Novel Variant in FOXC1 Associated with Atypical Axenfeld-Rieger Syndrome. *BMC Med. Genomics* **2021**, *14*, doi:10.1186/s12920-021-01130-7.

S15. Sinha, I.; Sinha-Hikim, A.P.; Wagers, A.J.; Sinha-Hikim, I. Testosterone Is Essential for Skeletal Muscle Growth in Aged Mice in a Heterochronic Parabiosis Model. *Cell Tissue Res.* **2014**, *357*, 815–821, doi:10.1007/s00441-014-1900-2.

S16. Liang, S.; Liu, D.; Xiao, Z.; Greenbaum, J.; Shen, H.; Xiao, H.; Deng, H. Repurposing Approved Drugs for Sarcopenia Based on Transcriptomics Data in Humans. *Pharmaceuticals* **2023**, *16*, doi:10.3390/ph16040607.

S17. Inoue, M.; Hayashi, A.; Taguchi, T.; Arai, R.; Sasaki, S.; Takano, K.; Inoue, Y.; Shichiri, M. Effects of Canagliflozin on Body Composition and Hepatic Fat Content in Type 2 Diabetes Patients with Non-Alcoholic Fatty Liver Disease. J. Diabetes Investig. 2019, 10, 1004–1011, doi:10.1111/jdi.12980.

S18. Wang, T.; Shao, W.; Huang, Z.; Tang, H.; Zhang, J.; Ding, Z.; Huang, K. MOGONET Integrates Multi-Omics Data Using Graph Convolutional Networks Allowing Patient Classification and Biomarker Identification. *Nat. Commun.* **2021**, *12*, doi:10.1038/s41467-021-23774-w.

S19. Elmarakeby, H.A.; Hwang, J.; Arafeh, R.; Crowdis, J.; Gang, S.; Liu, D.; AlDubayan, S.H.; Salari, K.; Kregel, S.; Richter, C.; et al. Biologically Informed Deep Neural Network for Prostate Cancer Discovery. *Nature* **2021**, *598*, 348–352, doi:10.1038/s41586-021-03922-4.

S20. Argelaguet, R.; Velten, B.; Arnol, D.; Dietrich, S.; Zenz, T.; Marioni, J.C.; Buettner, F.; Huber, W.; Stegle, O. Multi‐Omics Factor Analysis—a Framework for Unsupervised Integration of Multi‐omics Data Sets. Mol. Syst. Biol. 2018, 14, doi:10.15252/msb.20178124.

S21. Velten, B.; Braunger, J.M.; Argelaguet, R.; Arnol, D.; Wirbel, J.; Bredikhin, D.; Zeller, G.; Stegle, O. Identifying Temporal and Spatial Patterns of Variation from Multimodal Data Using MEFISTO. Nat. Methods 2022, 19, 179–186, doi:10.1038/s41592-021-01343-9.

S22. Su, W.M.; Gu, X.J.; Dou, M.; Duan, Q.Q.; Jiang, Z.; Yin, K.F.; Cai, W.C.; Cao, B.; Wang, Y.; Chen, Y.P. Systematic Druggable Genome-Wide Mendelian Randomisation Identifies Therapeutic Targets for Alzheimer’s Disease. *J. Neurol. Neurosurg. Psychiatry* **2023**, *94*, 954–961, doi:10.1136/jnnp-2023-331142.
